# A Clarification on Quantum‐Metric‐Induced Nonlinear Transport

**DOI:** 10.1002/advs.202514818

**Published:** 2025-11-23

**Authors:** Xiao‐Bin Qiang, Tianyu Liu, Zi‐Xuan Gao, Hai‐Zhou Lu, X. C. Xie

**Affiliations:** ^1^ State Key Laboratory of Quantum Functional Materials Department of Physics, and Guangdong Basic Research Center of Excellence for Quantum Science Southern University of Science and Technology (SUSTech) Shenzhen 518055 China; ^2^ Quantum Science Center of Guangdong‐Hong Kong‐Macao Greater Bay Area (Guangdong) Shenzhen 518045 China; ^3^ Shenzhen Institute for Quantum Science and Engineering and Department of Physics Southern University of Science and Technology (SUSTech) Shenzhen 518055 China; ^4^ Shenzhen Key Laboratory of Quantum Science and Engineering Shenzhen 518055 China; ^5^ International Center for Quantum Materials School of Physics Peking University Beijing 100871 China; ^6^ Interdisciplinary Center for Theoretical Physics and Information Sciences (ICTPIS) Fudan University Shanghai 200433 China; ^7^ Hefei National Laboratory Hefei 230088 China

**Keywords:** Berry connection polarizability, nonlinear transport, quantum geometry, quantum metric

## Abstract

Over the years, Berry curvature, which is associated with the imaginary part of the quantum geometric tensor, has profoundly impacted many branches of physics. Recently, quantum metric, the real part of the quantum geometric tensor, has been recognized as an indispensable part in comprehensively characterizing the intrinsic properties of condensed matter systems. The intrinsic second‐order nonlinear conductivity induced by the quantum metric has attracted significant recent interest. However, its expression varies across the literature. Here, this discrepancy is reconciled by systematically examining the nonlinear conductivity using the standard perturbation theory, the wave packet dynamics, and the Luttinger–Kohn approach. Moreover, inspired by the Dirac model, a toy model is proposed that suppresses the Berry‐curvature‐induced nonlinear transport, making it suitable for studying the quantum‐metric‐induced nonlinear conductivity. This work provides a clearer and more unified understanding of the quantum‐metric contribution to nonlinear transport. It also establishes a solid foundation for future theoretical developments and experimental explorations in this highly active and rapidly evolving field.

## Graphical abstract

How does the quantum metric truly govern the nonlinear transport? The longstanding theoretical discrepancies are resolved in quantum‐metric‐induced nonlinear transport and the correct intrinsic nonlinear conductivity is identified. Furthermore, a toy model is engineered to suppress the competing effects, uniquely highlighting the role of quantum metric and enabling the future detection of quantum metric in quantum materials.

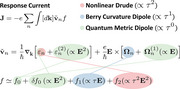

## 1 Introduction

Berry curvature^[^ ^1^ ^]^ characterizes the geometry of the Hilbert space and has significantly influenced the research paradigm of modern condensed matter physics.^[^ ^2^ ^]^ One prominent example is the family of quantum Hall effects. Both the integer quantum Hall effect^[^ ^3^ ^]^ and the quantum anomalous Hall effect^[^ ^4^ ^]^ originate from the quantized integral of Berry curvature in the Brillouin zone.^[^ ^5^ ^]^ The uniform distribution of Berry curvature in the Landau levels responsible for the fractional quantum Hall effect^[^ ^6^ ^]^ has made such uniformity a key design criterion for realizing the fractional quantum anomalous Hall effect,^[^ ^7^, ^8^, ^9^ ^]^ which is a candidate platform for hosting Fibonacci anyons suitable for universal topological quantum computation.^[^ ^10^ ^]^ In addition to various quantum Hall effects, Berry curvature also plays a key role in topological phases of matter,^[^ ^11^, ^12^ ^]^ orbital magnetization,^[^ ^13^, ^14^, ^15^ ^]^ and nonlinear transport.^[^ ^16^, ^17^, ^18^, ^19^, ^20^, ^21^ ^]^

Quantum metric is also a geometric measure and, together with Berry curvature, constitutes the quantum geometric tensor of Hilbert space.^[^ ^22^, ^23^, ^24^, ^25^, ^26^ ^]^ The quantum metric is critical for understanding flat‐band superconductivity,^[^ ^27^, ^28^, ^29^, ^30^, ^31^ ^]^ and is also essential for elucidating the fractional quantum anomalous Hall effect^[^ ^6^, ^8^, ^9^, ^32^ ^]^ and nonlinear transport.^[^ ^33^, ^34^, ^35^, ^36^ ^]^ Although analogous to Berry curvature in the context of nonlinear transport, it is crucial to note that quantum‐metric‐induced nonlinear transport is intrinsic (i.e., independent of the relaxation time).^[^ ^37^, ^38^, ^39^ ^]^ Such nonlinear transport has recently been proposed and identified in antiferromagnets, where both the inversion symmetry (P) and the time‐reversal symmetry (T) are broken but the combined PT symmetry is preserved (e.g., CuMnAs,^[^ ^33^ ^]^ Mn_2_Au,^[^ ^34^ ^]^ and MnBi_2_Te_4_^[^ ^35^, ^36^ ^]^), further demonstrating a broad relevance and growing impact of this topic.

Multiple theories have been proposed to elucidate the quantum‐metric‐induced nonlinear transport,^[^ ^37^, ^38^, ^39^ ^]^ with their predictions partly corroborated by transport experiments of MnBi_2_Te_4_.^[^ ^35^, ^36^ ^]^ However, there remains an inconsistency regarding the specific form of quantum‐metric‐induced nonlinear conductivity within these theories (see **Table** **1** for details). Moreover, the wave packet dynamics^[^ ^37^ ^]^ predicts the suppression of in‐plane nonlinear transport under any out‐of‐plane n‐fold rotational symmetry $C_{n}^{z}$ (n = 2, 3, 4, 6) and the absence of longitudinal response,^[^ ^33^, ^34^ ^]^ but the Luttinger–Kohn approach^[^ ^38^ ^]^ and the quantum kinetics^[^ ^39^ ^]^ are compatible with $C_{n}^{z}$ and longitudinal response. This discrepancy aggravates the existing confusion in understanding quantum‐metric‐induced nonlinear transport. Therefore, a clarification of the form of quantum‐metric‐induced nonlinear conductivity is aspired.

**Table 1:** Analytical expressions for the quantum‐metric‐induced second‐order nonlinear conductivity $\sigma_{\mathrm{ijk}}^{\mathrm{qm}}$, where indices i, j, k denote spatial directions. The results are derived using the wave packet dynamics (WPD), the Luttinger–Kohn approach (LKA), and the quantum kinetics (QK). Here, $\left[dk\right]=d^{d}k/{\left(2\pi \right)}^{d}$ with d being the dimension, $f_{0}=f_{0}\left(\varepsilon_{nk}\right)$ represents the Fermi‐Dirac distribution function evaluated at the unperturbed band energy $\varepsilon_{nk}$ for band index n and crystal momentum k, and $G_{n}=G_{n}\left(k\right)$ is the band‐normalized quantum metric tensor of the nth band.

**Theories**	**Expressions**
WPD^[^ ^37^ ^]^	$-\frac{e^{3}}{\hbar }\sum_{n}\int \left[dk\right]\left[\partial_{i}G_{n}^{\mathrm{jk}}-\frac{1}{2}\left(\partial_{k}G_{n}^{\mathrm{ij}}+\partial_{j}G_{n}^{\mathrm{ik}}\right)\right]f_{0}$
LKA^[^ ^38^ ^]^	$-\frac{e^{3}}{\hbar }\sum_{n}\int \left[dk\right]\left[2\partial_{i}G_{n}^{\mathrm{jk}}-\frac{1}{2}\left(\partial_{k}G_{n}^{\mathrm{ij}}+\partial_{j}G_{n}^{\mathrm{ik}}\right)\right]f_{0}$
QK^[^ ^39^ ^]^	$-\frac{e^{3}}{\hbar }\sum_{n}\int \left[dk\right]\left[\frac{1}{2}\partial_{i}G_{n}^{\mathrm{jk}}-\left(\partial_{k}G_{n}^{\mathrm{ij}}+\partial_{j}G_{n}^{\mathrm{ik}}\right)\right]f_{0}$

In this paper, we aim to reconcile existing theoretical formulations of quantum‐metric‐induced nonlinear conductivity. Treating the driving electric field as a perturbation, we first derive the electric‐field‐modified Berry connection ${\tilde{A}}_{n}\left(k\right)$ and band energy ${\tilde{\varepsilon }}_{nk}$ via the standard perturbation theory. A general expression for second‐order nonlinear conductivity is derived. We then elucidate that the same nonlinear conductivity can be essentially derived through the wave packet dynamics, which yields identical electric‐field‐modified Berry connection and band energy. Moreover, the nonlinear conductivity obtained from the Luttinger–Kohn approach can be made consistent with those arising from the standard perturbation theory and the wave packet dynamics. Lastly, based on symmetry considerations, we propose a toy model that suppresses the Berry‐curvature‐induced second‐order nonlinear transport, thereby highlighting the nonlinear transport resulting from the quantum metric.

## 2 Standard Perturbation Theory

For a system subject to a weak driving electric field E, the current density can be written as

(1) $$J=-e\sum_{n}\int \left[dk\right]{\tilde{v}}_{n}\left(k\right)f\left(k\right),$$

where e is the elementary charge, $\left[dk\right]\equiv d^{d}k/{\left(2\pi \right)}^{d}$ with d denoting the dimension, ${\tilde{v}}_{n}\left(k\right)$ is the electric‐field‐modified velocity of the nth energy band at crystal momentum k, and f(k) denotes the nonequilibrium distribution function. Specifically, the electric‐field‐modified velocity is given by ${\tilde{v}}_{n}\left(k\right)=\frac{1}{\hbar }\nabla_{k}{\tilde{\varepsilon }}_{nk}+\frac{e}{\hbar }E\times {\tilde{\Omega }}_{n}\left(k\right)$,^[^ ^2^ ^]^ where ${\tilde{\varepsilon }}_{nk}$ is the electric‐field‐modified energy of the nth band, and ${\tilde{\Omega }}_{n}\left(k\right)=\nabla_{k}\times {\tilde{A}}_{n}\left(k\right)$ represents the electric‐field‐modified Berry curvature of the nth energy band, arising from the electric‐field‐modified intraband Berry connection ${\tilde{A}}_{n}\left(k\right)$. On the other hand, under the relaxation time approximation, f(k) can be extracted through the Boltzmann equation as $f\left(k\right)=\sum_{\nu =0}^{\infty }{\left(\frac{\tau e}{\hbar }E\cdot \nabla_{k}\right)}^{\nu }f_{0}\left({\tilde{\varepsilon }}_{nk}\right)$,^[^ ^18^, ^40^, ^41^ ^]^ where τ is the relaxation time, and $f_{0}\left({\tilde{\varepsilon }}_{nk}\right)$ represents the Fermi‐Dirac distribution function evaluated at ${\tilde{\varepsilon }}_{nk}$. For a sufficiently weak E, the ith component of the response current [Equation (1)] can be approximated by a power series in E as

(2) $$J_{i}=\sigma_{ij}E_{j}+\sigma_{ijk}E_{j}E_{k}+\cdots ,$$

where $\sigma_{\mathrm{ij}}$ is the linear conductivity and $\sigma_{\mathrm{ijk}}$ is the second‐order nonlinear conductivity (indices i,j,k label spatial directions). According to Equation (1), the determination of $\sigma_{\mathrm{ijk}}$ requires expanding ${\tilde{v}}_{n}\left(k\right)$ and f(k) to the second order of E. Therefore, it would be sufficient to estimate ${\tilde{A}}_{n}\left(k\right)$ and ${\tilde{\varepsilon }}_{nk}$ to the first and second orders of E, respectively.

To estimate ${\tilde{A}}_{n}\left(k\right)$, we consider the real‐space Hamiltonian

(3) $$\hat{H}={\hat{H}}_{0}+eE\cdot \hat{r},$$

where ${\hat{H}}_{0}$ is the Hamiltonian characterizing a field‐free periodic system and $eE\cdot \hat{r}$ is a perturbation induced by a weak applied electric field E. The eigenvalue problem of ${\hat{H}}_{0}$ can be solved by Bloch's theorem with the eigenenergy denoted by $\varepsilon_{nk}$ and the eigenvector $|\psi_{nk}\left(r\right)\rangle =e^{ik\cdot r}\left|u_{nk}\left(r\right)\right\rangle$ comprised of a plane wave $e^{ik\cdot r}$ and a unit‐cell‐periodic part $|u_{nk}\left(r\right)\rangle$. The eigenvectors of $\hat{H}$ can then be perturbatively derived, to the first order of E, as $|{\tilde{\psi }}_{nk}\left(r\right)\rangle =|\psi_{nk}\left(r\right)\rangle +\frac{V}{{\left(2\pi \right)}^{d}}\int d^{d}k^{\prime }\sum_{m\neq n}\left\langle \psi_{mk^{\prime }}\left(r\right)\right|eE\cdot \hat{r}|\psi_{nk}\left(r\right)\rangle {\left(\varepsilon_{nk}-\varepsilon_{mk^{\prime }}\right)}^{-1}\left|\psi_{mk^{\prime }}\left(r\right)\right\rangle$. The unit‐cell‐periodic part of $|{\tilde{\psi }}_{nk}\left(r\right)\rangle$ consequently becomes (detailed derivations in the Supporting Information ^[^ ^42^ ^]^)

(4) $$|{\tilde{u}}_{nk}\left(r\right)\rangle =|u_{nk}\left(r\right)\rangle +\sum_{m\neq n}\frac{eE\cdot A_{\mathrm{mn}}\left(k\right)}{\varepsilon_{nk}-\varepsilon_{mk}}\left|u_{mk}\left(r\right)\right\rangle ,$$

where $A_{\mathrm{mn}}\left(k\right)=\langle u_{mk}\left(r\right)|i\nabla_{k}\left|u_{nk}\left(r\right)\right\rangle$ is the field‐free interband Berry connection. Accordingly, we can write the electric‐field‐modified Berry connection, to the first order of E, as

(5) $${\tilde{A}}_{n}\left(k\right)=\frac{\left\langle {\tilde{u}}_{nk}\left(r\right)|i\nabla_{k}|{\tilde{u}}_{nk}\left(r\right)\right\rangle }{\left\langle {\tilde{u}}_{nk}\left(r\right)|{\tilde{u}}_{nk}\left(r\right)\right\rangle }≃A_{n}\left(k\right)+G_{n}\left(k\right)\cdot E,$$

where $A_{n}\left(k\right)=\langle u_{nk}\left(r\right)|i\nabla_{k}\left|u_{nk}\left(r\right)\right\rangle$ is the field‐free Berry connection of the nth energy band^[^ ^2^ ^]^ and $G_{n}\left(k\right)=2e\text{Re}\sum_{m\neq n}A_{\mathrm{nm}}\left(k\right)A_{\mathrm{mn}}\left(k\right)/\left(\varepsilon_{nk}-\varepsilon_{mk}\right)$ is the Berry connection polarizability.^[^ ^37^ ^]^ It is worth noting that $G_{n}\left(k\right)$ is U(1)‐gauge invariant and the electric‐field‐induced Berry connection $G_{n}\left(k\right)\cdot E$ is an observable.^[^ ^42^ ^]^ The electric‐field‐induced Berry connection originates from the quantum metric, because $G_{n}\left(k\right)$ differs from the band‐normalized quantum metric $G_{n}\left(k\right)=2\text{Re}\sum_{m\neq n}A_{\mathrm{nm}}\left(k\right)A_{\mathrm{mn}}\left(k\right)/\left(\varepsilon_{nk}-\varepsilon_{mk}\right)$ only by a multiplicative factor of e,^[^ ^38^, ^39^ ^]^ and the latter is related to the quantum metric $g_{n}\left(k\right)=\text{Re}\sum_{m\neq n}A_{\mathrm{nm}}\left(k\right)A_{\mathrm{mn}}\left(k\right)$ ^[^ ^22^, ^23^, ^24^, ^25^, ^26^ ^]^ through $G_{n}\left(k\right)=-\partial g_{n}\left(k\right)/\partial \varepsilon_{nk}$.

The evaluation of ${\tilde{\varepsilon }}_{nk}$ in the context of the standard perturbation theory is a more subtle issue. Both the first‐order energy correction $\left\langle \psi_{nk}\left(r\right)|eE\cdot \hat{r}|\psi_{nk}\left(r\right)\right\rangle$ and the second‐order energy correction $\sum_{m\neq n}\langle \psi_{nk}\left(r\right)|eE\cdot \hat{r}\left|\psi_{mk}\left(r\right)\right\rangle \left[eE\cdot A_{\mathrm{mn}}\left(k\right)/\left(\varepsilon_{nk}-\varepsilon_{mk}\right)\right]$ formally diverge, because the matrix elements of $\hat{r}$ are not well‐defined in the basis of Bloch eigenvectors.^[^ ^42^ ^]^ To resolve the issue, we construct the following unit‐cell‐periodic Hamiltonian

(6) $$H\left(k\right)=H_{0}\left(k\right)+eE\cdot i\nabla_{k},$$

where $H_{0}\left(k\right)=e^{-ik\cdot r}{\hat{H}}_{0}e^{ik\cdot r}$ is the Bloch Hamiltonian. It is straightforward to find that the nth eigenvector of H(k), to the first order of E, coincides with Equation (4). This validates Equation (6) as an appropriate ansatz for describing a periodic system subject to an electric field. The perturbative energy corrections then become well‐defined and the electric‐field‐modified band energy, to the second order of E, reads^[^ ^42^ ^]^

(7) $${\tilde{\varepsilon }}_{nk}=\varepsilon_{nk}+\frac{e}{2}E\cdot G_{n}\left(k\right)\cdot E,$$

where we have neglected the first‐order energy correction $\langle u_{nk}\left(r\right)|eE\cdot i\nabla_{k}\left|u_{nk}\left(r\right)\right\rangle =eE\cdot A_{n}\left(k\right)$ because it depends on gauge and hence does not contribute to physical observables. We mention that the first‐order energy correction arises from the second term in Equation (6)—a regularization that overcomes the ill‐definedness of the position operator $\hat{r}$ and restores the translational symmetry, but inevitably introduces gauge dependence as $i\nabla_{k}\to i\nabla_{k}-\nabla_{k}\phi_{k}$, where $\phi_{k}$ is the phase of the chosen U(1) gauge.

Equations (5) and (7) allow for expanding ${\tilde{v}}_{n}\left(k\right)$ and f(k) to the second order of E. The ith component of the velocity, to the second order of E, is given by [42]

(8) $$\begin{array}{ccc} {\tilde{v}}_{n}^{i} & = & \frac{1}{\hbar }\partial_{i}\varepsilon_{n}+\frac{e}{2\hbar }\left(\Omega_{n}^{\mathrm{ij}}E_{j}+\Omega_{n}^{\mathrm{ik}}E_{k}\right)+\frac{e}{2\hbar }\left[3\partial_{i}G_{n}^{\mathrm{jk}}-\left(\partial_{j}G_{n}^{\mathrm{ik}}+\partial_{k}G_{n}^{\mathrm{ij}}\right)\right]E_{j}E_{k}, \end{array}$$

where the argument k is omitted to prevent misguidance in subscripts/superscripts (e.g., $\varepsilon_{nk}\to \varepsilon_{n}$ and $\partial_{k_{i}}\to \partial_{i}$) and Berry curvature tensor $\Omega_{n}^{ij}=\varepsilon_{ijk}\Omega_{n}^{k}=\partial_{i}A_{n}^{j}-\partial_{j}A_{n}^{i}$ is introduced to symmetrize indices {j,k}. On the other hand, the non‐equilibrium distribution function, to the second order of E, reads^[^ ^42^ ^]^

(9) $$f=f_{0}+\frac{e}{2}G_{n}^{ij}E_{i}E_{j}f_{0}^{\prime }+\frac{e\tau }{\hbar }E_{i}\partial_{i}f_{0}+\frac{e^{2}\tau^{2}}{\hbar^{2}}E_{i}E_{j}\partial_{i}\partial_{j}f_{0},$$

with $f=f\left(k\right),\;f_{0}=f_{0}\left(\varepsilon_{nk}\right)$, $f_{0}^{\prime }=\partial f_{0}\left(\varepsilon_{nk}\right)/\partial \varepsilon_{nk}$. The second term in Equation (9) is the electric‐field‐induced correction to the Fermi‐Dirac distribution function.

**Figure 1:**
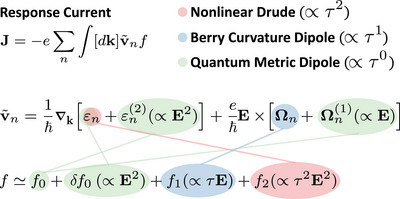
Distinct physical origins of the second‐order nonlinear conductivity. The contributions from the nonlinear Drude term, the Berry curvature dipole, and the quantum metric dipole are indicated in red, blue, and green, respectively. Note that E and $E^{2}$ are used to demonstrate the order of electric‐field‐induced perturbation, and the exact E dependences of the relevant quantities are given in Equations (5), (7), and (9).

Plugging Equations (8) and (9) into Equation (1), the second‐order nonlinear conductivity is obtained as [42]

(10) $$\sigma_{ijk}=\sigma_{ijk}^{d}+\sigma_{ijk}^{\text{bc}}+\sigma_{ijk}^{\text{qm}},$$

which originates from three distinct physical mechanisms, differentiated by their dependence on the relaxation time τ: the Drude term $\sigma_{ijk}^{d}$ exhibits a quadratic τ‐dependence, the Berry‐curvature‐induced term $\sigma_{ijk}^{\text{bc}}$ scales linearly with τ, and the quantum‐metric‐induced term $\sigma_{ijk}^{\text{qm}}$ is independent of τ, thus being an intrinsic property (see **Figure** **1**). The three contributions explicitly read

(11) $$\sigma_{\mathrm{ijk}}^{d}=-\frac{e^{3}\tau^{2}}{\hbar^{3}}\sum_{n}\int \left[dk\right]\left(\partial_{i}\partial_{j}\partial_{k}\varepsilon_{n}\right)f_{0},$$

(12) $$\sigma_{\mathrm{ijk}}^{\text{bc}}=\frac{e^{3}\tau }{2\hbar^{2}}\sum_{n}\int \left[dk\right]\left(\partial_{k}\Omega_{n}^{\mathrm{ij}}+\partial_{j}\Omega_{n}^{\mathrm{ik}}\right)f_{0},$$

(13) $$\sigma_{\mathrm{ijk}}^{\text{qm}}=-\frac{e^{3}}{\hbar }\sum_{n}\int \left[dk\right]\left[\partial_{i}G_{n}^{\mathrm{jk}}-\frac{1}{2}\left(\partial_{j}G_{n}^{\mathrm{ik}}+\partial_{k}G_{n}^{\mathrm{ij}}\right)\right]f_{0},$$

where the band‐normalized quantum metric is used in Equation (13) to facilitate comparison with existing theories (see **Table** 1). The quantum‐metric‐induced nonlinear conductivity [Equation (13)] constitutes the central result of the standard perturbation theory, consistent with that derived from the wave packet dynamics^[^ ^37^ ^]^ while distinct from those derived by the Luttinger–Kohn approach^[^ ^38^ ^]^ and the quantum kinetics.^[^ ^39^ ^]^ Alternatively, it can be reformulated in terms of $f_{0}^{\prime }$ as

(14) $$\sigma_{ijk}^{\text{qm}}=e^{3}\sum_{n}\int \left[dk\right]\Lambda_{n}^{ijk}f_{0}^{\prime },$$

where $\Lambda_{n}^{ijk}=v_{n}^{i}G_{n}^{jk}-\frac{1}{2}\left(v_{n}^{j}G_{n}^{ik}+v_{n}^{k}G_{n}^{ij}\right)$ is known as the quantum metric dipole. According to Equation (14), it is straightforward to check that the quantum‐metric‐induced nonlinear longitudinal transport is prohibited, as can be seen from the identity $\sigma_{iii}^{\text{qm}}=0$.

## 3 Wave Packet Dynamics

We now examine the consistency between the standard perturbation theory and the wave packet dynamics. Within the framework of the wave packet dynamics, the quantum state associated with the nth band of the Bloch Hamiltonian $H_{0}\left(k\right)$ is described by a wave packet $|W_{n}\left(r\right)\rangle =\int d^{d}kw_{n}\left(k\right)\left|\psi_{nk}\left(r\right)\right\rangle$, where the superposition weight $w_{n}\left(k\right)$ satisfies $|w_{n}{\left(k\right)|}^{2}=\frac{V}{{\left(2\pi \right)}^{d}}\delta \left(k-k_{c}\right)$ with $k_{c}$ labeling the momentum center of the wave packet.^[^ ^2^ ^]^ In the presence of an applied electric field, the wave packet develops interband mixing and is thus modified to [37]

(15) $$\begin{array}{cc} |{\tilde{W}}_{n}\left(r\right)\rangle = & \int d^{d}k\left(1-\delta \right)w_{n}\left(k\right)\left|\psi_{nk}\left(r\right)\right\rangle \\ & +\int d^{d}k\sum_{m\neq n}w_{m}^{\left(1\right)}\left(k\right)\left|\psi_{mk}\left(r\right)\right\rangle , \end{array}$$

where the coefficient $w_{m}^{\left(1\right)}\left(k\right)=w_{n}\left(k\right)eE\cdot A_{\mathrm{mn}}\left(k\right)/\left(\varepsilon_{nk}-\varepsilon_{mk}\right)$ represents the first‐order amplitude of the admixture from the mth band and the parameter $\delta =\frac{1}{2}\frac{{\left(2\pi \right)}^{d}}{V}\int d^{d}k\sum_{m\neq n}{\left|w_{m}^{\left(1\right)}\left(k\right)\right|}^{2}$ ensures the normalization of the modified wave packet.^[^ ^42^ ^]^

The electric‐field‐induced Berry connection can be extracted from the positional shift of the wave packet. Specifically, in the presence of the applied electric field, the wave packet center is located at [42]

(16) $$\langle {\tilde{W}}_{n}\left(r\right)|\hat{r}|{\tilde{W}}_{n}\left(r\right)\rangle =r_{c}+G_{n}\left(k_{c}\right)\cdot E,$$

where $r_{c}=\left\langle W_{n}\left(r\right)\right|\hat{r}\left|W_{n}\left(r\right)\right\rangle =-\nabla_{k}argw_{n}\left(k\right){|}_{k=k_{c}}+A_{n}\left(k_{c}\right)$ represents the field‐free wave packet center. The second term in Equation (16) can thus be understood as a correction to the Berry connection.^[^ ^37^ ^]^ Consequently, the electric‐field‐modified Berry connection reads ${\tilde{A}}_{n}\left(k_{c}\right)=A_{n}\left(k_{c}\right)+G_{n}\left(k_{c}\right)\cdot E$, which is consistent with Equation (5). On the other hand, the electric‐field‐modified band energy can be calculated by extracting the gauge invariant part of $\left\langle {\tilde{W}}_{n}\left(r\right)|\hat{H}|{\tilde{W}}_{n}\left(r\right)\right\rangle$ as ${\tilde{\varepsilon }}_{nk_{c}}=\varepsilon_{nk_{c}}+\frac{e}{2}E\cdot G_{n}\left(k_{c}\right)\cdot E$,^[^ ^42^ ^]^ which is consistent with Equation (7). As the second‐order nonlinear conductivity is fully determined by the electric‐field‐modified Berry connection (to the first order of E) and the electric‐field‐modified band energy (to the second order of E), the wave packet dynamics yields a nonlinear conductivity identical to that derived from the standard perturbation theory.

## 4 Luttinger–Kohn Approach

Beyond the standard perturbation theory and the wave packet dynamics, the electric‐field‐modified Berry connection and band energy can also be extracted using the Luttinger–Kohn approach.^[^ ^43^, ^44^ ^]^ To implement the approach, we begin by rewriting Equation (6) as

(17) $$H\left(k\right)=H_{0}\left(k\right)+\lambda eE\cdot i\nabla_{k},$$

where λ is introduced for transparency to track the order of perturbation and can be set to unity as needed. We then perform a unitary Schrieffer‐Wolff transformation $|u_{nk}\left(r\right)\rangle \to e^{\lambda S(k)}\left|u_{nk}\left(r\right)\right\rangle$, where the generator S(k) must be anti‐Hermitian to guarantee the unitarity of the transformation.^[^ ^44^ ^]^ One of the key findings of the Luttinger–Kohn approach is that an appropriate choice of the generator S(k) renders $e^{\lambda S(k)}\left|u_{nk}\left(r\right)\right\rangle$ an eigenvector of H(k) to the desired order^[^ ^43^, ^44^ ^]^ and the corresponding eigenenergies can be obtained from the eigenvalue problem of the transformed Hamiltonian $H_{\text{eff}}\left(k\right)=e^{-\lambda S(k)}H\left(k\right)e^{\lambda S(k)}$. To the second order of λ, the transformed Hamiltonian reads

(18) $$\begin{array}{cc} H_{\text{eff}}\left(k\right)=\; & H_{0}\left(k\right)+\lambda \left(eE\cdot i\nabla_{k}+\left[H_{0}\left(k\right),S\left(k\right)\right]\right)\\ & +\lambda^{2}\left(\left[eE\cdot i\nabla_{k},S\left(k\right)\right]+\frac{1}{2}\left[\left[H_{0}\left(k\right),S\left(k\right)\right],S\left(k\right)\right]\right). \end{array}$$

The goal here is to choose a generator that eliminates the off‐diagonal elements of the second term in Equation (18) such that $H_{\text{eff}}\left(k\right)$ becomes diagonalized to the first order of λ and the energy correction arising from the third term (scaled as $\lambda^{2}$) coincides with its expectation value in $|u_{nk}\left(r\right)\rangle$. The elimination condition, which is referred to as the Luttinger–Kohn condition, gives rise to the off‐diagonal elements of the generator as [42]

(19) $$S_{\mathrm{mn}}\left(k\right)=\frac{eE\cdot A_{\mathrm{mn}}\left(k\right)}{\varepsilon_{nk}-\varepsilon_{mk}},$$

where m≠n. It is worth noting that the diagonal entries of S(k) cannot be uniquely determined, though the anti‐Hermicity requires their real parts to vanish. Nevertheless, the nth eigenenergy of H(k) coincides with $\left\langle u_{nk}\left(r\right)|H_{\text{eff}}\left(k\right)|u_{nk}\left(r\right)\right\rangle$. Its gauge invariant part, at λ=1, corresponds to the electric‐field‐modified band energy and reads ${\tilde{\varepsilon }}_{nk}=\varepsilon_{nk}+\frac{e}{2}E\cdot G_{n}\left(k\right)\cdot E$,^[^ ^42^ ^]^ which is identical to Equation (7). With the Schrieffer–Wolff transformed unit‐cell‐periodic state $e^{S(k)}\left|u_{nk}\left(r\right)\right\rangle$, the electric‐field‐modified Berry connection reads ${\tilde{A}}_{n}\left(k\right)=\langle u_{nk}\left(r\right)|e^{-S(k)}i\nabla_{k}e^{S(k)}\left|u_{nk}\left(r\right)\right\rangle =A_{n}\left(k\right)+G_{n}\left(k\right)\cdot E$,^[^ ^42^ ^]^ which is identical to Equation (5). Since the Luttinger–Kohn approach produces the same electric‐field‐modified Berry connection and band energy as the standard perturbation theory, it is expected to yield an identical second‐order nonlinear conductivity.

## 5 Origin of Inconsistency

Our derivations using three distinct methods (i.e., the standard perturbation theory in Section 2, the wave packet dynamics in Section 3, and the Luttinger–Kohn approach in Section 4) agree with Ref. [37] but are inconsistent with Refs. [38] and [39]. Herein, we briefly discuss the origin of the inconsistency concerning the form of the quantum‐metric‐induced nonlinear conductivity.

### 5.1 Luttinger–Kohn Approach

We note that our derivations in Section 4 differ from those of Ref. [38] in both the electric‐field‐modified band energy and the argument of the distribution function.

In the Luttinger–Kohn approach of Ref. [38], the variation of band energy due to the applied electric field E (cf. Equation (5) of Ref. [38]) is attributed solely to the electric‐field‐induced Berry connection $G_{n}\left(k\right)\cdot E$. However, it is crucial to recognize that the bare band energy itself must also be renormalized by the electric field, because the wave function [Equation (4)] now acquires  dependence. Taking into account the E dependence, the bare band energy becomes $\langle {\tilde{u}}_{nk}\left(r\right)|H_{0}\left(k\right)\left|{\tilde{u}}_{nk}\left(r\right)\right\rangle /\left\langle {\tilde{u}}_{nk}\left(r\right)|{\tilde{u}}_{nk}\left(r\right)\right\rangle =\varepsilon_{nk}-\frac{e}{2}E\cdot G_{n}\left(k\right)\cdot E$, which yields the correct electric‐field‐modified band energy [Equation (7)] when combined with the Berry connection contribution $E\cdot G_{n}\left(k\right)\cdot E$.

When solving for the nonlinear conductivity within the Boltzmann formalism, Ref. [38] evaluates the Fermi–Dirac distribution function at the unrenormalized bare band energy $\varepsilon_{nk}$. While this is justified in the context of linear response theory as the first‐order energy correction $eE\cdot A_{n}\left(k\right)$ depends on gauge and should not affect the observable physics, it is essential to expand the Fermi–Dirac distribution function to the second order of E when deriving the quantum‐metric‐induced nonlinear conductivity. Consequently, it is more appropriate to express the Fermi–Dirac distribution function as $f_{0}\left({\tilde{\varepsilon }}_{nk}\right)=f_{0}\left(\varepsilon_{nk}\right)+\frac{e}{2}E\cdot G_{n}\left(k\right)\cdot E\frac{\partial f_{0}}{\partial \varepsilon_{nk}}$, which incorporates an additional Fermi‐sea occupation correction quadratic in E.^[^ ^45^, ^46^, ^47^ ^]^

### 5.2 Quantum Kinetics

Regarding the quantum‐metric‐induced nonlinear conductivity derived from quantum kinetics,^[^ ^39^ ^]^ the discrepancy in the expression (see **Table** 1) likely has a more complex and subtle origin. Although we do not attempt to reproduce the full density matrix formalism of Ref. [39], we note that a so‐called *modified scattering time* τ/2, where τ is the regular scattering time, is adopted when evaluating certain density matrix contributions. This modification of the relaxation time seems not adequately justified and may compromise the reliability of the derived quantum‐metric‐induced nonlinear conductivity (**Table** 1), which indeed differs from ours [Equation (13)].

## 6 Toy Model for Quantum‐Metric‐Induced Nonlinear Transport

We now construct a toy—though not necessarily minimal—model that is suitable for investigating the quantum‐metric‐induced second‐order nonlinear transport. For simplicity, we restrict the analysis to a planar geometry such that only nonlinear Hall conductivities $\sigma_{xyy}^{\text{qm}}$ and $\sigma_{yxx}^{\text{qm}}$ are relevant. Symmetry analysis indicates that the emergence of nonlinear Hall conductivities requires the breaking of $C_{n}^{z}$, P, and T symmetries.^[^ ^34^ ^]^ In addition, PT symmetry is preferred because it suppresses the nonlinear Hall effect arising from the Berry curvature dipole, rendering the quantum‐metric‐dipole contribution dominant. With all these requirements, we propose the following Bloch Hamiltonian

(20) $$H_{0}\left(k\right)=v\tau_{x}\left(k_{x}\sigma_{x}+k_{y}\sigma_{y}\right)+\left(m-bk^{2}\right)\tau_{z}+tk_{x}\tau_{z},$$

where v, m, b and t are model parameters; and σ and τ are Pauli matrices. The first two terms in Equation (20) constitute a Dirac model with PT symmetry,^[^ ^48^ ^]^ whereas the last term, while preserving the PT symmetry, breaks $C_{n}^{z}$, P, and T symmetries.

**Figure 2:**
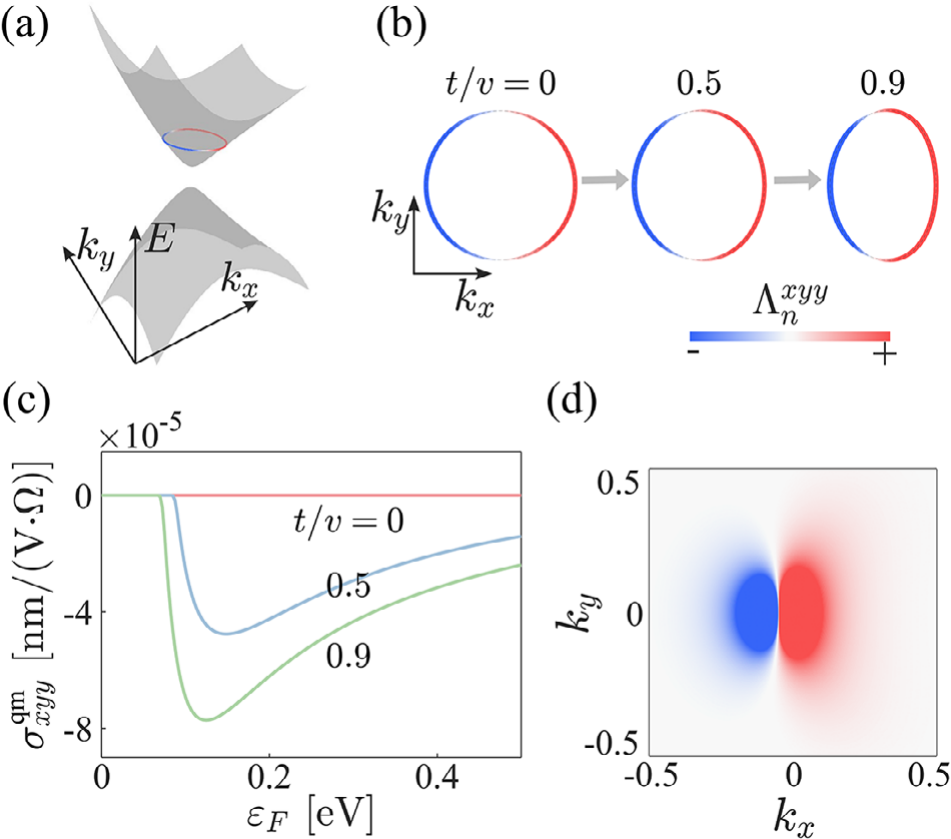
Theoretical results from the toy model [Equation (20)]. a) Band structure at t/v = 0.9 with the Fermi surface placed at $\varepsilon_{F}$ = 0.25 eV. b) Fermi surfaces at $\varepsilon_{F}$ = 0.25 eV for t/v = 0, 0.5, 0.9. The color scale indicates the magnitude of the quantum metric dipole $\Lambda_{n}^{xyy}$. c) Calculated nonlinear Hall conductivity $\sigma_{yxx}^{\text{qm}}$ as a function of the Fermi energy $\varepsilon_{F}$. d) Momentum‐resolved distribution of $\Lambda_{n}^{xyy}$ for t/v = 0.9. The model parameters used are v = 1 eV·nm, m = 0.1 eV, and b = 1 eV·nm^2^.

The Hamiltonian exhibits a deformed gapped Dirac cone band structure $\varepsilon_{k}=\pm \sqrt{v^{2}k^{2}+{\left(m-bk^{2}+tk_{x}\right)}^{2}}$ (**Figure** **2**a), where the parameter t measures the degree of deformation. The evolution of the Fermi surface with respect to t is illustrated in **Figure** 2b. For t=0, the symmetry‐breaking term vanishes, resulting in a symmetric distribution of the quantum metric dipole $\Lambda_{n}^{xyy}$ on the Fermi surface (**Figure** 2b) and a vanishing quantum‐metric‐induced nonlinear Hall conductivity $\sigma_{xyy}^{\text{qm}}$ (red line, **Figure** 2c). As t increases, the asymmetry in the distribution of $\Lambda_{n}^{xyy}$ grows (**Figure** 2b), leading to enhanced nonlinear Hall conductivities (blue and green curves, **Figure** 2c). To better visualize the asymmetry of $\Lambda_{n}^{xyy}$ across the Fermi surface, **k**‐resolved $\Lambda_{n}^{xyy}$ is shown in **Figure** 2d.

## 7 Conclusion

We have clarified the discrepancies among several existing theories for quantum‐metric‐induced second‐order nonlinear transport by examining the nonlinear conductivity using three distinct approaches: the standard perturbation theory, the wave packet dynamics, and the Luttinger–Kohn approach. Through careful symmetry design, we have proposed a PT‐symmetric toy model that suppresses the Berry‐curvature‐induced nonlinear Hall effect while allowing the quantum‐metric‐induced contribution to dominate. The magnitude of the resulting quantum‐metric‐induced nonlinear Hall conductivity positively correlates with the degree of breaking of rotational, inversion, and time‐reversal symmetries. This work not only clarifies the inconsistency in the theory of nonlinear transport but also proposes a suitable model for studying the quantum‐metric‐induced nonlinear Hall effect, which can potentially be realized in quantum materials. Furthermore, our framework can be extended to incorporate disorder effects and higher‐order quantum geometric contributions, paving the way for a more comprehensive understanding of nonlinear transport phenomena.

*Note added.—*When preparing the revised manuscript, we became aware of a related preprint,^[^ ^49^ ^]^ in which the interband quantum‐metric‐induced nonlinear conductivity is consistent with our Equation (13).

## Conflict of Interest

The authors declare no conflict of interest.

## Supporting information

Supporting Information

## Data Availability

The data that support the findings of this study are available from the corresponding author upon reasonable request.
