# Supplementary material for: A Clarification on Quantum‐Metric‐Induced Nonlinear Transport
Source: Adv Sci (Weinh). 2025 Nov 23;13(7):e14818. doi: 10.1002/advs.202514818 (PMC12866866; doi:10.1002/advs.202514818)
Supplement: Supplementary file 1 — Supporting Information [file ADVS-13-e14818-s001.pdf]

Supplemental Material for
"A Clarification on Quantum-Metric-Induced Nonlinear Transport"

Xiao-Bin Qiang,[1, 2, *] Tianyu Liu,[3, 4, *] Zi-Xuan Gao,[1, 2, *] Hai-Zhou Lu,[1, 2, †] and X. C. Xie[5, 6, 7]

[1] *State Key Laboratory of Quantum Functional Materials, Department of Physics, and Guangdong Basic Research Center of Excellence for Quantum Science, Southern University of Science and Technology (SUSTech), Shenzhen 518055, China*

[2] *Quantum Science Center of Guangdong-Hong Kong-Macao Greater Bay Area (Guangdong), Shenzhen 518045, China*

[3] *International Quantum Academy, Shenzhen 518048, China*

[4] *Shenzhen Key Laboratory of Quantum Science and Engineering, Shenzhen 518055, China*

[5] *International Center for Quantum Materials, School of Physics, Peking University, Beijing 100871, China*

[6] *Institute for Nanoelectronic Devices and Quantum Computing, Fudan University, Shanghai 200433, China*

[7] *Hefei National Laboratory, Hefei 230088, China*

(Dated: November 17, 2025)

CONTENTS

SI. Standard Perturbation Theory S1

SII. Wave Packet Dynamics S3
- A. Wave Packet Construction S3
- B. Positional Shift S5
- C. Wave Packet Energy S6

SIII. Luttinger-Kohn Approach S7

SIV. Nonlinear Transport S8
- A. Velocity S8
- B. Boltzmann Formalism S9
- C. Nonlinear Conductivity S10

References S11

SI. STANDARD PERTURBATION THEORY

We now explicitly derive the electric-field-modified Berry connection and band energy using the standard perturbation theory. For a generic periodic system subject to an electric field, the Hamiltonian operator can be written as

$$\hat{\mathcal{H}} = \hat{\mathcal{H}}_0 + e\mathbf{E}\cdot\hat{\mathbf{r}}, \tag{S1}$$

where $\hat{\mathcal{H}}_0$ is the periodic Hamiltonian operator defined in the real space and $e\mathbf{E}\cdot\hat{\mathbf{r}}$ is the electric-field-induced potential with $\hat{\mathbf{r}}$ representing the position operator. For a sufficiently weak electric field $\mathbf{E}$, the eigenvalue problem of $\hat{\mathcal{H}}$ can be solved by perturbatively correcting the eigenenergy and eigenvector of $\hat{\mathcal{H}}_0$, respectively denoted as $\varepsilon_{n\mathbf{k}}$ and $|\psi_{n\mathbf{k}}(\mathbf{r})\rangle$, where $|\psi_{n\mathbf{k}}(\mathbf{r})\rangle$ characterizes a Bloch wave. In the context of the standard perturbation theory, the eigenvector, to the first order of $\mathbf{E}$, reads

$$\left|\tilde{\psi}_{n\mathbf{k}}(\mathbf{r})\right\rangle = |\psi_{n\mathbf{k}}(\mathbf{r})\rangle + \frac{V}{(2\pi)^d}\int d^d\mathbf{k}' \sum_{m\neq n} \frac{\langle\psi_{m\mathbf{k}'}(\mathbf{r})|e\mathbf{E}\cdot\hat{\mathbf{r}}|\psi_{n\mathbf{k}}(\mathbf{r})\rangle}{\varepsilon_{n\mathbf{k}}-\varepsilon_{m\mathbf{k}'}}|\psi_{m\mathbf{k}'}(\mathbf{r})\rangle. \tag{S2}$$

---

* These authors contributed equally to this work.

† Corresponding author: luhz@sustech.edu.cn

Bloch's theorem requires the eigenvector of $\hat{\mathcal{H}}_0$ to be written as $|\psi_{n\mathbf{k}}(\mathbf{r})\rangle = \mathrm{e}^{\mathrm{i}\mathbf{k}\cdot\mathbf{r}}|u_{n\mathbf{k}}(\mathbf{r})\rangle$, where $|u_{n\mathbf{k}}(\mathbf{r})\rangle$ is defined in a unit cell and has the periodicity of the Bravais lattice. The numerator in the second term in Eq. (S2) can be evaluated by using

$$\begin{aligned}\langle\psi_{m\mathbf{k}'}(\mathbf{r})|\hat{\mathbf{r}}|\psi_{n\mathbf{k}}(\mathbf{r})\rangle &= \int d^d\mathbf{r}u^*_{m\mathbf{k}'}(\mathbf{r})\mathrm{e}^{-\mathrm{i}\mathbf{k}'\cdot\mathbf{r}}\mathbf{r}\mathrm{e}^{\mathrm{i}\mathbf{k}\cdot\mathbf{r}}u_{n\mathbf{k}}(\mathbf{r}) = -\mathrm{i}\int d^d\mathbf{r}u^*_{m\mathbf{k}'}(\mathbf{r})\mathrm{e}^{-\mathrm{i}\mathbf{k}'\cdot\mathbf{r}}(\nabla_{\mathbf{k}}\mathrm{e}^{\mathrm{i}\mathbf{k}\cdot\mathbf{r}})u_{n\mathbf{k}}(\mathbf{r})\\ &= -\mathrm{i}\nabla_{\mathbf{k}}\int d^d\mathbf{r}u^*_{m\mathbf{k}'}(\mathbf{r})\mathrm{e}^{-\mathrm{i}\mathbf{k}'\cdot\mathbf{r}}\mathrm{e}^{\mathrm{i}\mathbf{k}\cdot\mathbf{r}}u_{n\mathbf{k}}(\mathbf{r}) + \int d^d\mathbf{r}\mathrm{e}^{\mathrm{i}(\mathbf{k}-\mathbf{k}')\cdot\mathbf{r}}u^*_{m\mathbf{k}'}(\mathbf{r})\mathrm{i}\nabla_{\mathbf{k}}u_{n\mathbf{k}}(\mathbf{r})\\ &= -\mathrm{i}\nabla_{\mathbf{k}}\langle\psi_{m\mathbf{k}'}(\mathbf{r})|\psi_{n\mathbf{k}}(\mathbf{r})\rangle + \sum_{\mathbf{R}}\mathrm{e}^{\mathrm{i}(\mathbf{k}-\mathbf{k}')\cdot\mathbf{R}}\int_{\mathrm{u.c.}} d^d\mathbf{r}\mathrm{e}^{\mathrm{i}(\mathbf{k}-\mathbf{k}')\cdot\mathbf{r}}u^*_{m\mathbf{k}'}(\mathbf{r})\mathrm{i}\nabla_{\mathbf{k}}u_{n\mathbf{k}}(\mathbf{r})\\ &= \frac{(2\pi)^d}{V}\delta(\mathbf{k}-\mathbf{k}')\int_{\mathrm{u.c.}} d^d\mathbf{r}\mathrm{e}^{\mathrm{i}(\mathbf{k}-\mathbf{k}')\cdot\mathbf{r}}u^*_{m\mathbf{k}'}(\mathbf{r})\mathrm{i}\nabla_{\mathbf{k}}u_{n\mathbf{k}}(\mathbf{r}),\end{aligned} \tag{S3}$$

where the integration over real space is conducted by first integrating over a unit cell and then summing over all unit cells (parameterized by the Bravais lattice vector $\mathbf{R}$), i.e., $\int d^d\mathbf{r} = \sum_{\mathbf{R}}\int_{\mathrm{u.c.}} d^d\mathbf{r}$. When deriving Eq. (S3), we have used the Poisson summation formula $\sum_{\mathbf{R}}\mathrm{e}^{\mathrm{i}(\mathbf{k}-\mathbf{k}')\cdot\mathbf{R}} = \frac{(2\pi)^d}{V}\delta(\mathbf{k}-\mathbf{k}')$, where $\delta(\mathbf{k}-\mathbf{k}')$ is the Dirac delta function and V is the volume of the unit cell. Note that the orthonormalization of the Bloch wave functions $\langle\psi_{m\mathbf{k}'}(\mathbf{r})|\psi_{n\mathbf{k}}(\mathbf{r})\rangle = \frac{(2\pi)^d}{V}\delta_{mn}\delta(\mathbf{k}-\mathbf{k}')$ can be straightforwardly derived using the Poisson summation formula and $\langle u_{m\mathbf{k}}(\mathbf{r})|u_{n\mathbf{k}}(\mathbf{r})\rangle = \delta_{mn}$, where δ_{mn} is the Kronecker delta. Plugging Eq. (S3) into Eq. (S2), the electric-field-modified eigenvector reads

$$\begin{aligned}\left|\tilde{\psi}_{n\mathbf{k}}(\mathbf{r})\right\rangle &= |\psi_{n\mathbf{k}}(\mathbf{r})\rangle + e\mathbf{E}\cdot\frac{V}{(2\pi)^d}\int d^d\mathbf{k}'\sum_{m\neq n}\frac{(2\pi)^d}{V}\delta(\mathbf{k}-\mathbf{k}')\int_{\mathrm{u.c.}} d^d\mathbf{r}\mathrm{e}^{\mathrm{i}(\mathbf{k}-\mathbf{k}')\cdot\mathbf{r}}u^*_{m\mathbf{k}'}(\mathbf{r})\mathrm{i}\nabla_{\mathbf{k}}u_{n\mathbf{k}}(\mathbf{r})\frac{|\psi_{m\mathbf{k}'}(\mathbf{r})\rangle}{\varepsilon_{n\mathbf{k}}-\varepsilon_{m\mathbf{k}'}}\\ &= |\psi_{n\mathbf{k}}(\mathbf{r})\rangle + e\mathbf{E}\cdot\sum_{m\neq n}\int_{\mathrm{u.c.}} d^d\mathbf{r}u^*_{m\mathbf{k}}(\mathbf{r})\mathrm{i}\nabla_{\mathbf{k}}u_{n\mathbf{k}}(\mathbf{r})\frac{|\psi_{m\mathbf{k}}(\mathbf{r})\rangle}{\varepsilon_{n\mathbf{k}}-\varepsilon_{m\mathbf{k}}}\\ &= |\psi_{n\mathbf{k}}(\mathbf{r})\rangle + \sum_{m\neq n}\frac{e\mathbf{E}\cdot\boldsymbol{\mathcal{A}}_{mn}(\mathbf{k})}{\varepsilon_{n\mathbf{k}}-\varepsilon_{m\mathbf{k}}}|\psi_{m\mathbf{k}}(\mathbf{r})\rangle,\end{aligned} \tag{S4}$$

where $\boldsymbol{\mathcal{A}}_{mn}(\mathbf{k}) = \int_{\mathrm{u.c.}} d^d\mathbf{r}u^*_{m\mathbf{k}}(\mathbf{r})\mathrm{i}\nabla_{\mathbf{k}}u_{n\mathbf{k}}(\mathbf{r})$ is the interband Berry connection. The unit-cell-periodic part of $|\tilde{\psi}_{n\mathbf{k}}(\mathbf{r})\rangle$ can be extracted as

$$|\tilde{u}_{n\mathbf{k}}(\mathbf{r})\rangle = |u_{n\mathbf{k}}(\mathbf{r})\rangle + \sum_{m\neq n}\frac{e\mathbf{E}\cdot\boldsymbol{\mathcal{A}}_{mn}(\mathbf{k})}{\varepsilon_{n\mathbf{k}}-\varepsilon_{m\mathbf{k}}}|u_{m\mathbf{k}}(\mathbf{r})\rangle. \tag{S5}$$

It is critically important to note that such an electric-field-modified unit-cell-periodic state has not yet been normalized.

We now calculate the electric-field-modified Berry connection for the nth energy band. The nth intraband Berry connection, to the first order of $\mathbf{E}$, becomes

$$\begin{aligned}\tilde{\boldsymbol{\mathcal{A}}}_n(\mathbf{k}) &= \frac{\langle\tilde{u}_{n\mathbf{k}}(\mathbf{r})|\mathrm{i}\nabla_{\mathbf{k}}|\tilde{u}_{n\mathbf{k}}(\mathbf{r})\rangle}{\langle\tilde{u}_{n\mathbf{k}}(\mathbf{r})|\tilde{u}_{n\mathbf{k}}(\mathbf{r})\rangle} = \langle u_{n\mathbf{k}}(\mathbf{r})|\mathrm{i}\nabla_{\mathbf{k}}|u_{n\mathbf{k}}(\mathbf{r})\rangle + \left[\sum_{m\neq n}\frac{e\mathbf{E}\cdot\boldsymbol{\mathcal{A}}_{mn}(\mathbf{k})}{\varepsilon_{n\mathbf{k}}-\varepsilon_{m\mathbf{k}}}\langle u_{n\mathbf{k}}(\mathbf{r})|\mathrm{i}\nabla_{\mathbf{k}}|u_{m\mathbf{k}}(\mathbf{r})\rangle + \mathrm{c.c.}\right]\\ &= \boldsymbol{\mathcal{A}}_n(\mathbf{k}) + \left[e\sum_{m\neq n}\frac{\boldsymbol{\mathcal{A}}_{nm}(\mathbf{k})\boldsymbol{\mathcal{A}}_{mn}(\mathbf{k})}{\varepsilon_{n\mathbf{k}}-\varepsilon_{m\mathbf{k}}}\cdot\mathbf{E} + \mathrm{c.c.}\right] = \boldsymbol{\mathcal{A}}_n(\mathbf{k}) + 2e\,\mathrm{Re}\sum_{m\neq n}\frac{\boldsymbol{\mathcal{A}}_{nm}(\mathbf{k})\boldsymbol{\mathcal{A}}_{mn}(\mathbf{k})}{\varepsilon_{n\mathbf{k}}-\varepsilon_{m\mathbf{k}}}\cdot\mathbf{E}\\ &= \boldsymbol{\mathcal{A}}_n(\mathbf{k}) + \mathbf{G}_n(\mathbf{k})\cdot\mathbf{E},\end{aligned} \tag{S6}$$

where $\mathbf{G}_n(\mathbf{k}) = 2e\,\mathrm{Re}\sum_{m\neq n}\frac{\boldsymbol{\mathcal{A}}_{nm}(\mathbf{k})\boldsymbol{\mathcal{A}}_{mn}(\mathbf{k})}{\varepsilon_{n\mathbf{k}}-\varepsilon_{m\mathbf{k}}}$ is the Berry connection polarizability tensor, a gauge invariant quantity [1]. Equation (S6) is identical to Eq. (5) in the main text.

The evaluation of the electric-field-modified band energy is more subtle. In the context of the standard perturbation theory, the first- and second-order energy corrections respectively read

$$\begin{aligned}\varepsilon^{(1)}_{n\mathbf{k}} &= e\mathbf{E}\cdot\langle\psi_{n\mathbf{k}}(\mathbf{r})|\hat{\mathbf{r}}|\psi_{n\mathbf{k}}(\mathbf{r})\rangle,\\ \varepsilon^{(2)}_{n\mathbf{k}} &= \sum_{m\neq n}\frac{e\mathbf{E}\cdot\boldsymbol{\mathcal{A}}_{mn}(\mathbf{k})}{\varepsilon_{n\mathbf{k}}-\varepsilon_{m\mathbf{k}}}e\mathbf{E}\cdot\langle\psi_{n\mathbf{k}}(\mathbf{r})|\hat{\mathbf{r}}|\psi_{m\mathbf{k}}(\mathbf{r})\rangle.\end{aligned} \tag{S7}$$

It is straightforward to find that both corrections diverge because $\hat{\mathbf{r}}$ is not a legitimate operator in a periodic system [2]. To circumvent the divergence, we examine the electric-field-modified unit-cell-periodic state $|\tilde{u}_{n\mathbf{k}}(\mathbf{r})\rangle$. Inspired by the form of Eq. (S5), we construct a momentum-space Hamiltonian

$$\mathcal{H}(\mathbf{k}) = \mathcal{H}_0(\mathbf{k}) + e\mathbf{E}\cdot \mathrm{i}\nabla_{\mathbf{k}}, \tag{S8}$$

where $\mathcal{H}_0(\mathbf{k}) = \mathrm{e}^{-\mathrm{i}\mathbf{k}\cdot\mathbf{r}}\hat{\mathcal{H}}_0\mathrm{e}^{\mathrm{i}\mathbf{k}\cdot\mathbf{r}}$ is the Bloch Hamiltonian with eigenenergy $\varepsilon_{n\mathbf{k}}$ and eigenvector $|u_{n\mathbf{k}}(\mathbf{r})\rangle$. From the perspective of the standard perturbation theory, it is straightforward to find that the eigenvector of $\mathcal{H}(\mathbf{k})$, to the first order of $\mathbf{E}$, coincides with Eq. (S5). This suggests Eq. (S8) as a valid ansatz for the periodic Hamiltonian in the momentum space [3], where the perturbation $e\mathbf{E}\cdot\hat{\mathbf{r}}$ is regularized to $e\mathbf{E}\cdot \mathrm{i}\nabla_{\mathbf{k}}$, though $\hat{\mathbf{r}} \neq \mathrm{e}^{\mathrm{i}\mathbf{k}\cdot\mathbf{r}}\mathrm{i}\nabla_{\mathbf{k}}\mathrm{e}^{-\mathrm{i}\mathbf{k}\cdot\mathbf{r}}$ in the mathematical sense. It is worth noting that the energy corrections become well-defined with the help of $\mathcal{H}(\mathbf{k})$. Specifically, the first-order energy correction reads

$$\varepsilon^{(1)}_{n\mathbf{k}} = e\mathbf{E}\cdot\langle u_{n\mathbf{k}}(\mathbf{r})|\mathrm{i}\nabla_{\mathbf{k}}|u_{n\mathbf{k}}(\mathbf{r})\rangle = e\mathbf{E}\cdot\boldsymbol{\mathcal{A}}_n(\mathbf{k}), \tag{S9}$$

which converges but is not gauge invariant due to the appearance of the Berry connection $\boldsymbol{\mathcal{A}}_n(\mathbf{k})$. The second-order energy correction reads

$$\varepsilon^{(2)}_{n\mathbf{k}} = \sum_{m\neq n}\frac{|\langle u_{n\mathbf{k}}(\mathbf{r})|e\mathbf{E}\cdot\mathrm{i}\nabla_{\mathbf{k}}|u_{m\mathbf{k}}(\mathbf{r})\rangle|^2}{\varepsilon_{n\mathbf{k}}-\varepsilon_{m\mathbf{k}}} = e^2\sum_{m\neq n}\frac{\mathbf{E}\cdot\boldsymbol{\mathcal{A}}_{nm}(\mathbf{k})\boldsymbol{\mathcal{A}}^*_{nm}(\mathbf{k})\cdot\mathbf{E}}{\varepsilon_{n\mathbf{k}}-\varepsilon_{m\mathbf{k}}} = \frac{e}{2}\mathbf{E}\cdot\mathbf{G}_n(\mathbf{k})\cdot\mathbf{E}, \tag{S10}$$

where we have used $\boldsymbol{\mathcal{A}}^*_{nm}(\mathbf{k}) = -\mathrm{i}\langle u_{n\mathbf{k}}(\mathbf{r})|\nabla_{\mathbf{k}}u_{m\mathbf{k}}(\mathbf{r})\rangle^* = -\mathrm{i}\langle\nabla_{\mathbf{k}}u_{m\mathbf{k}}(\mathbf{r})|u_{n\mathbf{k}}(\mathbf{r})\rangle = \langle u_{m\mathbf{k}}(\mathbf{r})|\mathrm{i}\nabla_{\mathbf{k}}|u_{n\mathbf{k}}(\mathbf{r})\rangle = \boldsymbol{\mathcal{A}}_{mn}(\mathbf{k})$ and $|\mathbf{E}\cdot\boldsymbol{\mathcal{A}}_{mn}(\mathbf{k})|^2 = \mathbf{E}\cdot\mathrm{Re}[\boldsymbol{\mathcal{A}}_{nm}(\mathbf{k})\boldsymbol{\mathcal{A}}_{mn}(\mathbf{k})]\cdot\mathbf{E}$. Unlike $\varepsilon^{(1)}_{n\mathbf{k}}$, $\varepsilon^{(2)}_{n\mathbf{k}}$ possesses gauge invariance, which is inherited from the Berry connection polarizability tensor [1]. Consequently, the electric-field-modified band energy should be written as

$$\tilde{\varepsilon}_{n\mathbf{k}} = \varepsilon_{n\mathbf{k}} + \varepsilon^{(2)}_{n\mathbf{k}} = \varepsilon_{n\mathbf{k}} + \frac{e}{2}\mathbf{E}\cdot\mathbf{G}_n(\mathbf{k})\cdot\mathbf{E}, \tag{S11}$$

which is gauge invariant and observable. Equation (S11) is identical to Eq. (7) in the main text.

SII. WAVE PACKET DYNAMICS

We now derive the electric-field-modified Berry connection and band energy in the framework of the wave packet dynamics. We will start with a general formalism on wave packet construction and then investigate the effect of the electric field on the Berry connection and band energy.

A. Wave Packet Construction

For a wave packet centered at $\mathbf{r}_c$ with a narrow spatial spread, it would be reasonable to rearrange the model Hamiltonian to

$$\hat{\mathcal{H}} = \hat{\mathcal{H}}_c + e\mathbf{E}\cdot(\hat{\mathbf{r}}-\mathbf{r}_c), \tag{S12}$$

where $\hat{\mathcal{H}}_c = \hat{\mathcal{H}}_0 + e\mathbf{E}\cdot\mathbf{r}_c$ is the local Hamiltonian "felt" by the wave packet and $e\mathbf{E}\cdot(\hat{\mathbf{r}}-\mathbf{r}_c)$ is the electric-field-induced perturbation to the wave packet. As $e\mathbf{E}\cdot\mathbf{r}_c$ is a constant, $\hat{\mathcal{H}}_c$ and $\hat{\mathcal{H}}_0$ exhibit the same periodicity and share the same Bloch eigenvectors. Explicitly, the eigenvalue equation of $\hat{\mathcal{H}}_c$ reads

$$\hat{\mathcal{H}}_c|\psi_{n\mathbf{k}}(\mathbf{r})\rangle = \varepsilon^c_{n\mathbf{k}}|\psi_{n\mathbf{k}}(\mathbf{r})\rangle, \tag{S13}$$

where $\varepsilon^c_{n\mathbf{k}} = \varepsilon_{n\mathbf{k}} + e\mathbf{E}\cdot\mathbf{r}_c$ is the nth eigenenergy. The wave packet can be conveniently constructed in terms of the Bloch eigenvectors as

$$|W_n(\mathbf{r})\rangle = \int d^d\mathbf{k}\, w_n(\mathbf{k})|\psi_{n\mathbf{k}}(\mathbf{r})\rangle, \tag{S14}$$

where $w_n(\mathbf{k})$ represents the superposition weight. The normalization of the wave packet requires

$$\begin{aligned}\langle W_n(\mathbf{r})|W_n(\mathbf{r})\rangle &= \int d^d\mathbf{k}\int d^d\mathbf{k}'\, w^*_n(\mathbf{k})w_n(\mathbf{k}')\langle\psi_{n\mathbf{k}}(\mathbf{r})|\psi_{n\mathbf{k}'}(\mathbf{r})\rangle = \frac{(2\pi)^d}{V}\int d^d\mathbf{k}\int d^d\mathbf{k}'\, w^*_n(\mathbf{k})w_n(\mathbf{k}')\delta(\mathbf{k}-\mathbf{k}')\\ &= \frac{(2\pi)^d}{V}\int d^d\mathbf{k}|w_n(\mathbf{k})|^2 = 1,\end{aligned} \tag{S15}$$

which suggests that the superposition weight satifies $|w_n(\mathbf{k})|^2 = \frac{V}{(2\pi)^d}\delta(\mathbf{k}-\mathbf{k}_c)$, with $\mathbf{k}_c$ labeling the momentum center of the wave packet.

In the presence of the electric-field-induced perturbation, the wave packet can develop mixing with other bands labeled by $m \neq n$ and is thus modified to

$$\left|\tilde{W}_n(\mathbf{r})\right\rangle = \int d^d\mathbf{k} \left[(1-\delta)w_n(\mathbf{k})|\psi_{n\mathbf{k}}(\mathbf{r})\rangle + \sum_{m\neq n} w_m^{(1)}(\mathbf{k})|\psi_{m\mathbf{k}}(\mathbf{r})\rangle\right], \tag{S16}$$

where the parameter δ and the coefficient $w_m^{(1)}(\mathbf{k})$ quantify the interband mixing. The normalization of the modified wave packet requires $\delta = \frac{1}{2}\frac{(2\pi)^d}{V}\int d^d\mathbf{k}\sum_{m\neq n}|w_m^{(1)}(\mathbf{k})|^2$. To determine $w_m^{(1)}(\mathbf{k})$, we require $|\tilde{W}_n(\mathbf{r})\rangle$ to be an eigenvector of $\hat{\mathcal{H}}$. We then multiply $\langle\psi_{l\neq n\mathbf{k}}(\mathbf{r})|$ to the eigenvalue equation and get

$$\left\langle\psi_{l\mathbf{k}}(\mathbf{r})\middle|\hat{\mathcal{H}}\middle|\tilde{W}_n(\mathbf{r})\right\rangle = \tilde{\varepsilon}_{n\mathbf{k}}\left\langle\psi_{l\mathbf{k}}(\mathbf{r})\middle|\tilde{W}_n(\mathbf{r})\right\rangle. \tag{S17}$$

The left-hand side of Eq. (S17), to the first order of $\mathbf{E}$, reads

$$\begin{aligned}
\left\langle\psi_{l\mathbf{k}}(\mathbf{r})\middle|\hat{\mathcal{H}}\middle|\tilde{W}_n(\mathbf{r})\right\rangle &= \int d^d\mathbf{k}'\left[\langle\psi_{l\mathbf{k}}(\mathbf{r})|(\hat{\mathcal{H}}_0 + e\mathbf{E}\cdot\hat{\mathbf{r}})(1-\delta)w_n(\mathbf{k}')|\psi_{n\mathbf{k}'}(\mathbf{r})\rangle + \sum_{m\neq n}\langle\psi_{l\mathbf{k}}(\mathbf{r})|(\hat{\mathcal{H}}_0 + e\mathbf{E}\cdot\hat{\mathbf{r}})w_m^{(1)}(\mathbf{k}')|\psi_{m\mathbf{k}'}(\mathbf{r})\rangle\right] \\
&\simeq \int d^d\mathbf{k}' w_n(\mathbf{k}')e\mathbf{E}\cdot\langle\psi_{l\mathbf{k}}(\mathbf{r})|\hat{\mathbf{r}}|\psi_{n\mathbf{k}'}(\mathbf{r})\rangle + \int d^d\mathbf{k}'\sum_{m\neq n}\varepsilon_{l\mathbf{k}}w_m^{(1)}(\mathbf{k}')\langle\psi_{l\mathbf{k}}(\mathbf{r})|\psi_{m\mathbf{k}'}(\mathbf{r})\rangle \\
&= \frac{(2\pi)^d}{V}\int d^d\mathbf{k}'\delta(\mathbf{k}-\mathbf{k}')w_n(\mathbf{k}')e\mathbf{E}\cdot\int_{\mathrm{u.c.}} d^d\mathbf{r}e^{\mathrm{i}(\mathbf{k}'-\mathbf{k})\cdot\mathbf{r}}u_{l\mathbf{k}}^*(\mathbf{r})\mathrm{i}\nabla_{\mathbf{k}'}u_{n\mathbf{k}'}(\mathbf{r}) + \frac{(2\pi)^d}{V}\varepsilon_{l\mathbf{k}}w_l^{(1)}(\mathbf{k}) \\
&= \frac{(2\pi)^d}{V}w_n(\mathbf{k})e\mathbf{E}\cdot\int_{\mathrm{u.c.}} d^d\mathbf{r}u_{l\mathbf{k}}^*(\mathbf{r})\mathrm{i}\nabla_{\mathbf{k}}u_{n\mathbf{k}}(\mathbf{r}) + \frac{(2\pi)^d}{V}\varepsilon_{l\mathbf{k}}w_l^{(1)}(\mathbf{k}) \\
&= \frac{(2\pi)^d}{V}\left[w_n(\mathbf{k})e\mathbf{E}\cdot\boldsymbol{\mathcal{A}}_{ln}(\mathbf{k}) + \varepsilon_{l\mathbf{k}}w_l^{(1)}(\mathbf{k})\right],
\end{aligned} \tag{S18}$$

where we have used Eq. (S3) and $\langle\psi_{m\mathbf{k}'}(\mathbf{r})|\psi_{n\mathbf{k}}(\mathbf{r})\rangle = \frac{(2\pi)^d}{V}\delta_{mn}\delta(\mathbf{k}-\mathbf{k}')$. In the meanwhile, the right-hand side of Eq. (S17), to the first order of $\mathbf{E}$, reads

$$\begin{aligned}
\tilde{\varepsilon}_{n\mathbf{k}}\left\langle\psi_{l\mathbf{k}}(\mathbf{r})\middle|\tilde{W}_n(\mathbf{r})\right\rangle &= \tilde{\varepsilon}_{n\mathbf{k}}\int d^d\mathbf{k}'\left[\langle\psi_{l\mathbf{k}}(\mathbf{r})|\psi_{n\mathbf{k}'}(\mathbf{r})\rangle(1-\delta)w_n(\mathbf{k}') + \sum_{m\neq n}\langle\psi_{l\mathbf{k}}(\mathbf{r})|\psi_{m\mathbf{k}'}(\mathbf{r})\rangle w_m^{(1)}(\mathbf{k}')\right] \\
&= \frac{(2\pi)^d}{V}\tilde{\varepsilon}_{n\mathbf{k}}\int d^d\mathbf{k}'\delta(\mathbf{k}-\mathbf{k}')\sum_{m\neq n}\delta_{lm}w_m^{(1)}(\mathbf{k}') \\
&\simeq \frac{(2\pi)^d}{V}\varepsilon_{n\mathbf{k}}w_l^{(1)}(\mathbf{k}).
\end{aligned} \tag{S19}$$

Making use of Eqs. (S18) and (S19), it is straightforward to find that

$$w_l^{(1)}(\mathbf{k}) = \frac{e\mathbf{E}\cdot\boldsymbol{\mathcal{A}}_{ln}(\mathbf{k})}{\varepsilon_{n\mathbf{k}} - \varepsilon_{l\mathbf{k}}}w_n(\mathbf{k}). \tag{S20}$$

The wave packet can then be explicitly constructed as

$$\left|\tilde{W}_n(\mathbf{r})\right\rangle = \int d^d\mathbf{k}\left[(1-\delta)w_n(\mathbf{k})|\psi_{n\mathbf{k}}(\mathbf{r})\rangle + \sum_{m\neq n}\mathcal{C}_{mn}(\mathbf{k})w_n(\mathbf{k})|\psi_{m\mathbf{k}}(\mathbf{r})\rangle\right], \tag{S21}$$

where we have defined the coefficient $\mathcal{C}_{mn}(\mathbf{k}) = \frac{e\mathbf{E}\cdot\boldsymbol{\mathcal{A}}_{mn}(\mathbf{k})}{\varepsilon_{n\mathbf{k}}-\varepsilon_{m\mathbf{k}}}$. We note that matrix $\mathcal{C}(\mathbf{k})$ is anti-Hermitian as $\mathcal{C}_{mn}(\mathbf{k})^* = \frac{e\mathbf{E}\cdot\boldsymbol{\mathcal{A}}_{mn}^*(\mathbf{k})}{\varepsilon_{n\mathbf{k}}-\varepsilon_{m\mathbf{k}}} = -\frac{e\mathbf{E}\cdot\boldsymbol{\mathcal{A}}_{nm}(\mathbf{k})}{\varepsilon_{m\mathbf{k}}-\varepsilon_{n\mathbf{k}}} = -\mathcal{C}_{nm}(\mathbf{k})$. To the first order of $\mathbf{E}$, the wave packet becomes unnormalized and reads

$$\left|\tilde{W}_n(\mathbf{r})\right\rangle \simeq \int d^d\mathbf{k}w_n(\mathbf{k})\left[|\psi_{n\mathbf{k}}(\mathbf{r})\rangle + \sum_{m\neq n}\frac{e\mathbf{E}\cdot\boldsymbol{\mathcal{A}}_{mn}(\mathbf{k})}{\varepsilon_{n\mathbf{k}}-\varepsilon_{m\mathbf{k}}}|\psi_{m\mathbf{k}}(\mathbf{r})\rangle\right] = \int d^d\mathbf{k}w_n(\mathbf{k})\left|\tilde{\psi}_{n\mathbf{k}}(\mathbf{r})\right\rangle, \tag{S22}$$

which is an analog to the field-free wave packet [Eq. (S14)] with the electric-field-modified Bloch state $|\tilde{\psi}_{n\mathbf{k}}(\mathbf{r})\rangle$ [Eq. (S4)] taking over the role of the Bloch eigenvector $|\psi_{n\mathbf{k}}(\mathbf{r})\rangle$.

B. Positional Shift

Because of the interband mixing, the real-space center of the wave packet should also shift accordingly. The field-free wave packet is centered at

$$\begin{aligned}\mathbf{r}_c = \langle W_n(\mathbf{r})|\hat{\mathbf{r}}|W_n(\mathbf{r})\rangle =& \int d^d\mathbf{k}\int d^d\mathbf{k}' w_n^*(\mathbf{k}')w_n(\mathbf{k})\langle\psi_{n\mathbf{k}'}(\mathbf{r})|\hat{\mathbf{r}}|\psi_{n\mathbf{k}}(\mathbf{r})\rangle \\ =& -\mathrm{i}\int d^d\mathbf{k}\int d^d\mathbf{k}' w_n^*(\mathbf{k}')w_n(\mathbf{k})\nabla_{\mathbf{k}}\langle\psi_{n\mathbf{k}'}(\mathbf{r})|\psi_{n\mathbf{k}}(\mathbf{r})\rangle \\ &+\frac{(2\pi)^d}{V}\int d^d\mathbf{k}\int d^d\mathbf{k}' w_n^*(\mathbf{k}')w_n(\mathbf{k})\delta(\mathbf{k}-\mathbf{k}')\int_{\mathrm{u.c.}} d^d\mathbf{r}\mathrm{e}^{\mathrm{i}(\mathbf{k}-\mathbf{k}')\cdot\mathbf{r}}u_{n\mathbf{k}'}^*(\mathbf{r})\mathrm{i}\nabla_{\mathbf{k}}u_{n\mathbf{k}}(\mathbf{r}),\end{aligned} \tag{S23}$$

where we have used Eq. (S3). The first term in Eq. (S23) can be further simplified by integrating by parts as

$$\begin{aligned}&-\mathrm{i}\int d^d\mathbf{k}\int d^d\mathbf{k}' w_n^*(\mathbf{k}')w_n(\mathbf{k})\nabla_{\mathbf{k}}\langle\psi_{n\mathbf{k}'}(\mathbf{r})|\psi_{n\mathbf{k}}(\mathbf{r})\rangle = \mathrm{i}\int d^d\mathbf{k}\int d^d\mathbf{k}' w_n^*(\mathbf{k}')[\nabla_{\mathbf{k}}w_n(\mathbf{k})]\langle\psi_{n\mathbf{k}'}(\mathbf{r})|\psi_{n\mathbf{k}}(\mathbf{r})\rangle \\ &=\frac{(2\pi)^d}{V}\mathrm{i}\int d^d\mathbf{k}\int d^d\mathbf{k}' w_n^*(\mathbf{k}')[\nabla_{\mathbf{k}}w_n(\mathbf{k})]\delta(\mathbf{k}-\mathbf{k}') = \frac{(2\pi)^d}{V}\mathrm{i}\int d^d\mathbf{k}w_n^*(\mathbf{k})[\nabla_{\mathbf{k}}w_n(\mathbf{k})] \\ &=\frac{(2\pi)^d}{V}\mathrm{i}\int d^d\mathbf{k}|w_n(\mathbf{k})|\mathrm{e}^{-\mathrm{i}\arg w_n(\mathbf{k})}\left[\mathrm{e}^{\mathrm{i}\arg w_n(\mathbf{k})}\nabla_{\mathbf{k}}|w_n(\mathbf{k})| + \mathrm{i}\nabla_{\mathbf{k}}\arg w_n(\mathbf{k})\mathrm{e}^{\mathrm{i}\arg w_n(\mathbf{k})}|w_n(\mathbf{k})|\right] \\ &=-\frac{(2\pi)^d}{V}\int d^d\mathbf{k}|w_n(\mathbf{k})|^2\nabla_{\mathbf{k}}\arg w_n(\mathbf{k}) = -\int d^d\mathbf{k}\delta(\mathbf{k}-\mathbf{k}_c)\nabla_{\mathbf{k}}\arg w_n(\mathbf{k}) \\ &=-\nabla_{\mathbf{k}}\arg w_n(\mathbf{k})|_{\mathbf{k}=\mathbf{k}_c},\end{aligned} \tag{S24}$$

where we resort to the facts that $\int d^d\mathbf{k}\nabla_{\mathbf{k}}[w_n(\mathbf{k})\langle\psi_{n\mathbf{k}'}(\mathbf{r})|\psi_{n\mathbf{k}}(\mathbf{r})\rangle] = 0$ and $\int d^d\mathbf{k}\nabla_{\mathbf{k}}|w_n(\mathbf{k})|^2 = 0$. In the meanwhile, the second term in Eq. (S23) reads

$$\begin{aligned}&\frac{(2\pi)^d}{V}\int d^d\mathbf{k}\int d^d\mathbf{k}' w_n^*(\mathbf{k}')w_n(\mathbf{k})\delta(\mathbf{k}-\mathbf{k}')\int_{\mathrm{u.c.}} d^d\mathbf{r}\mathrm{e}^{\mathrm{i}(\mathbf{k}-\mathbf{k}')\cdot\mathbf{r}}u_{nk'}^*(\mathbf{r})\mathrm{i}\nabla_{\mathbf{k}}u_{n\mathbf{k}}(\mathbf{r}) \\ &=\frac{(2\pi)^d}{V}\int d^d\mathbf{k}|w_n(\mathbf{k})|^2\int_{\mathrm{u.c.}} d^d\mathbf{r}u_{nk}^*(\mathbf{r})\mathrm{i}\nabla_{\mathbf{k}}u_{n\mathbf{k}}(\mathbf{r}) = \int d^d\mathbf{k}\delta(\mathbf{k}-\mathbf{k}_c)\boldsymbol{\mathcal{A}}_n(\mathbf{k}) \\ &=\boldsymbol{\mathcal{A}}_n(\mathbf{k}_c).\end{aligned} \tag{S25}$$

Therefore, the real-space center of the field-free wave packet is

$$\mathbf{r}_c = -\nabla_{\mathbf{k}}\arg w_n(\mathbf{k})|_{\mathbf{k}=\mathbf{k}_c} + \boldsymbol{\mathcal{A}}_n(\mathbf{k}_c). \tag{S26}$$

It is worth noting that $\mathbf{r}_c$ depends on gauge. On the one hand, although $|w_n(\mathbf{k})|^2 = \frac{V}{(2\pi)^d}\delta(\mathbf{k}-\mathbf{k}_c)$, there is no requirement on $\arg w_n(\mathbf{k})$, which can be chosen arbitrarily. On the other hand, the Berry connection also depends on gauge.

In the presence of the applied electric field, the real-space center of the wave packet should in general acquire a shift. The new center is located, to the first order of $\mathbf{E}$, at

$$\begin{aligned}\tilde{\mathbf{r}}_c = \left\langle\tilde{W}_n(\mathbf{r})\middle|\hat{\mathbf{r}}\middle|\tilde{W}_n(\mathbf{r})\right\rangle \simeq& \langle W_n(\mathbf{r})|\hat{\mathbf{r}}|W_n(\mathbf{r})\rangle + \left[\int d^d\mathbf{k}\sum_{m\neq n}\frac{e\mathbf{E}\cdot\boldsymbol{\mathcal{A}}_{mn}(\mathbf{k})}{\varepsilon_{n\mathbf{k}}-\varepsilon_{m\mathbf{k}}}w_n(\mathbf{k})\langle W_n(\mathbf{r})|\mathbf{r}|\psi_{m\mathbf{k}}(\mathbf{r})\rangle + \mathrm{c.c.}\right] \\ =\mathbf{r}_c + &\left[\int d^d\mathbf{k}\int d^d\mathbf{k}'\sum_{m\neq n}\frac{e\mathbf{E}\cdot\boldsymbol{\mathcal{A}}_{mn}(\mathbf{k})}{\varepsilon_{n\mathbf{k}}-\varepsilon_{m\mathbf{k}}}w_n^*(\mathbf{k}')w_n(\mathbf{k})\langle\psi_{n\mathbf{k}'}(\mathbf{r})|\mathbf{r}|\psi_{m\mathbf{k}}(\mathbf{r})\rangle + \mathrm{c.c.}\right]\end{aligned}$$

$$
\begin{aligned}
&= \mathbf{r}_c + \frac{(2\pi)^d}{V}\left[\int d^d\mathbf{k}\int d^d\mathbf{k}'\sum_{m\neq n}\frac{e\mathbf{E}\cdot\boldsymbol{\mathcal{A}}_{mn}(\mathbf{k})}{\varepsilon_{n\mathbf{k}}-\varepsilon_{m\mathbf{k}}}w_n^*(\mathbf{k}')w_n(\mathbf{k})\delta(\mathbf{k}-\mathbf{k}')\int_{\text{u.c.}} d^d\mathbf{r}e^{\mathrm{i}(\mathbf{k}-\mathbf{k}')\cdot\mathbf{r}}u^*_{n\mathbf{k}'}(\mathbf{r})\mathrm{i}\nabla_{\mathbf{k}}u_{m\mathbf{k}}(\mathbf{r}) + \text{c.c.}\right]\\
&= \mathbf{r}_c + \frac{(2\pi)^d}{V}\left[\int d^d\mathbf{k}\sum_{m\neq n}\frac{e\mathbf{E}\cdot\boldsymbol{\mathcal{A}}_{mn}(\mathbf{k})}{\varepsilon_{n\mathbf{k}}-\varepsilon_{m\mathbf{k}}}w_n^*(\mathbf{k})w_n(\mathbf{k})\int_{\text{u.c.}} d\mathbf{r}u^*_{n\mathbf{k}}(\mathbf{r})\mathrm{i}\nabla_{\mathbf{k}}u_{m\mathbf{k}}(\mathbf{r}) + \text{c.c.}\right]\\
&= \mathbf{r}_c + \left[\int d^d\mathbf{k}\sum_{m\neq n}\frac{e\mathbf{E}\cdot\boldsymbol{\mathcal{A}}_{mn}(\mathbf{k})}{\varepsilon_{n\mathbf{k}}-\varepsilon_{m\mathbf{k}}}\delta(\mathbf{k}-\mathbf{k}_c)\boldsymbol{\mathcal{A}}_{nm}(\mathbf{k}) + \text{c.c.}\right] = \mathbf{r}_c + \left[\int d^d\mathbf{k}\sum_{m\neq n}\frac{e\boldsymbol{\mathcal{A}}_{nm}(\mathbf{k})\boldsymbol{\mathcal{A}}_{mn}(\mathbf{k})\cdot\mathbf{E}}{\varepsilon_{n\mathbf{k}}-\varepsilon_{m\mathbf{k}}}\delta(\mathbf{k}-\mathbf{k}_c) + \text{c.c.}\right]\\
&= \mathbf{r}_c + \left[2e\,\mathrm{Re}\sum_{m\neq n}\frac{\boldsymbol{\mathcal{A}}_{nm}(\mathbf{k}_c)\boldsymbol{\mathcal{A}}_{mn}(\mathbf{k}_c)}{\varepsilon_{n\mathbf{k}_c}-\varepsilon_{m\mathbf{k}_c}}\right]\cdot\mathbf{E} = \mathbf{r}_c + \mathbf{G}_n(\mathbf{k}_c)\cdot\mathbf{E}\\
&= -\nabla_{\mathbf{k}}\arg w_n(\mathbf{k})|_{\mathbf{k}=\mathbf{k}_c} + \boldsymbol{\mathcal{A}}_n(\mathbf{k}_c) + \mathbf{G}_n(\mathbf{k}_c)\cdot\mathbf{E}.
\end{aligned}\tag{S27}
$$

According to Eq. (S27), it is straightforward to find that the applied electric field shifts the real-space center of the wave packet by $\tilde{\mathbf{r}}_c - \mathbf{r}_c = \mathbf{G}_n(\mathbf{k}_c)\cdot\mathbf{E}$. Although both $\tilde{\mathbf{r}}_c$ and $\mathbf{r}_c$ depend on gauge, the positional shift itself is indeed gauge invariant because of the appearance of Berry connection polarizability [1]. We choose a specific gauge by performing $|u_{n\mathbf{k}}(\mathbf{r})\rangle \to e^{-\mathrm{i}\arg w_n(\mathbf{k})}|u_{n\mathbf{k}}(\mathbf{r})\rangle$. The Berry connection is then transformed according to $\boldsymbol{\mathcal{A}}_n(\mathbf{k}) \to \boldsymbol{\mathcal{A}}_n(\mathbf{k}) + \nabla_{\mathbf{k}}\arg w_n(\mathbf{k})$, and the real-space centers are located at $\mathbf{r}_c = \boldsymbol{\mathcal{A}}_n(\mathbf{k}_c)$ and $\tilde{\mathbf{r}}_c = \boldsymbol{\mathcal{A}}_n(\mathbf{k}_c) + \mathbf{G}_n(\mathbf{k}_c)\cdot\mathbf{E}$. Therefore, the positional shift can be treated as a correction to the Berry connection. The electric-field-modified Berry connection then reads

$$
\tilde{\boldsymbol{\mathcal{A}}}_n(\mathbf{k}_c) = \boldsymbol{\mathcal{A}}_n(\mathbf{k}_c) + \mathbf{G}_n(\mathbf{k}_c)\cdot\mathbf{E}. \tag{S28}
$$

This expression is consistent with Eq. (S6) except that the argument here is the momentum center of the wave packet $\mathbf{k}_c = \int d^d\mathbf{k}|w_n(\mathbf{k})|^2\mathbf{k}$ rather than the crystal momentum $\mathbf{k}$.

C. Wave Packet Energy

We now calculate the energy of the wave packet. The contribution from the local Hamiltonian, to the second order of $\mathbf{E}$, reads

$$
\begin{aligned}
\left\langle\tilde{W}_n(\mathbf{r})\middle|\hat{\mathcal{H}}_c\middle|\tilde{W}_n(\mathbf{r})\right\rangle &= \int d^d\mathbf{k}\int d^d\mathbf{k}'(1-\delta)^2 w_n^*(\mathbf{k}')w_n(\mathbf{k})\left\langle\psi_{n\mathbf{k}'}(\mathbf{r})\middle|\hat{\mathcal{H}}_c\middle|\psi_{n\mathbf{k}}(\mathbf{r})\right\rangle\\
&\quad+\int d^d\mathbf{k}\int d^d\mathbf{k}'\sum_{l\neq n}\sum_{m\neq n}[w_l^{(1)}(\mathbf{k}')]^*w_m^{(1)}(\mathbf{k})\left\langle\psi_{l\mathbf{k}'}(\mathbf{r})\middle|\hat{\mathcal{H}}_c\middle|\psi_{m\mathbf{k}}(\mathbf{r})\right\rangle\\
&=\frac{(2\pi)^d}{V}\int d^d\mathbf{k}\int d^d\mathbf{k}'\delta(\mathbf{k}-\mathbf{k}')\left[(1-\delta)^2w_n^*(\mathbf{k}')w_n(\mathbf{k})\varepsilon^c_{n\mathbf{k}} + \sum_{l\neq n}\sum_{m\neq n}[w_l^{(1)}(\mathbf{k}')]^*w_m^{(1)}(\mathbf{k})\delta_{lm}\varepsilon^c_{m\mathbf{k}}\right]\\
&=\frac{(2\pi)^d}{V}\int d^d\mathbf{k}\left[(1-\delta)^2|w_n(\mathbf{k})|^2\varepsilon^c_{n\mathbf{k}} + \sum_{m\neq n}|w_m^{(1)}(\mathbf{k})|^2\varepsilon^c_{m\mathbf{k}}\right]\\
&=(1-\delta)^2\int d^d\mathbf{k}\delta(\mathbf{k}-\mathbf{k}_c)\varepsilon^c_{n\mathbf{k}} + \frac{(2\pi)^d}{V}\int d^d\mathbf{k}\sum_{m\neq n}|w_m^{(1)}(\mathbf{k})|^2\varepsilon^c_{m\mathbf{k}}\\
&=\left[1-\frac{(2\pi)^d}{V}\int d^d\mathbf{k}\sum_{m\neq n}|w_m^{(1)}(\mathbf{k})|^2\right]\varepsilon^c_{n\mathbf{k}_c} + \frac{(2\pi)^d}{V}\int d^d\mathbf{k}\sum_{m\neq n}|w_m^{(1)}(\mathbf{k})|^2\varepsilon^c_{m\mathbf{k}}\\
&=\varepsilon^c_{n\mathbf{k}_c} - \frac{(2\pi)^d}{V}\int d^d\mathbf{k}\sum_{m\neq n}\left|\frac{e\mathbf{E}\cdot\boldsymbol{\mathcal{A}}_{mn}(\mathbf{k})}{\varepsilon_{n\mathbf{k}}-\varepsilon_{m\mathbf{k}}}\right|^2|w_n(\mathbf{k})|^2(\varepsilon^c_{n\mathbf{k}_c}-\varepsilon^c_{m\mathbf{k}})\\
&=\varepsilon^c_{n\mathbf{k}_c} - \int d^d\mathbf{k}\delta(\mathbf{k}-\mathbf{k}_c)\sum_{m\neq n}\left|\frac{e\mathbf{E}\cdot\boldsymbol{\mathcal{A}}_{mn}(\mathbf{k})}{\varepsilon_{n\mathbf{k}}-\varepsilon_{m\mathbf{k}}}\right|^2(\varepsilon^c_{n\mathbf{k}_c}-\varepsilon^c_{m\mathbf{k}})
\end{aligned}
$$

$$
\begin{aligned}
&=\varepsilon^c_{n\mathbf{k}_c} - \sum_{m\neq n}\left|\frac{e\mathbf{E}\cdot\boldsymbol{\mathcal{A}}_{mn}(\mathbf{k}_c)}{\varepsilon_{n\mathbf{k}_c}-\varepsilon_{m\mathbf{k}_c}}\right|^2(\varepsilon^c_{n\mathbf{k}_c}-\varepsilon^c_{m\mathbf{k}_c}) \simeq \varepsilon^c_{n\mathbf{k}_c} - e^2\sum_{m\neq n}\frac{|\mathbf{E}\cdot\boldsymbol{\mathcal{A}}_{mn}(\mathbf{k}_c)|^2}{\varepsilon_{n\mathbf{k}_c}-\varepsilon_{m\mathbf{k}_c}} \\
&=\varepsilon_{n\mathbf{k}_c} + e\mathbf{E}\cdot\mathbf{r}_c - e^2\mathbf{E}\cdot\sum_{m\neq n}\frac{\mathrm{Re}[\boldsymbol{\mathcal{A}}_{nm}(\mathbf{k}_c)\boldsymbol{\mathcal{A}}_{mn}(\mathbf{k}_c)]}{\varepsilon_{n\mathbf{k}_c}-\varepsilon_{m\mathbf{k}_c}}\cdot\mathbf{E} \\
&=\varepsilon_{n\mathbf{k}_c} + e\mathbf{E}\cdot\mathbf{r}_c - \frac{e}{2}\mathbf{E}\cdot\mathbf{G}_n(\mathbf{k}_c)\cdot\mathbf{E}, \qquad \text{(S29)}
\end{aligned}
$$

where we notice the fact that $|\mathbf{E}\cdot\boldsymbol{\mathcal{A}}_{mn}(\mathbf{k}_c)|^2 = \mathbf{E}\cdot\mathrm{Re}[\boldsymbol{\mathcal{A}}_{nm}(\mathbf{k}_c)\boldsymbol{\mathcal{A}}_{mn}(\mathbf{k}_c)]\cdot\mathbf{E}$. In the meanwhile, the contribution from the perturbation reads

$$
\left\langle\tilde{W}_n(\mathbf{r})\middle|\mathbf{E}\cdot(\hat{\mathbf{r}}-\mathbf{r}_c)\middle|\tilde{W}_n(\mathbf{r})\right\rangle = e\mathbf{E}\cdot\left[\left\langle\tilde{W}_n(\mathbf{r})\middle|\hat{\mathbf{r}}\middle|\tilde{W}_n(\mathbf{r})\right\rangle - \mathbf{r}_c\right] = e\mathbf{E}\cdot\mathbf{G}_n(\mathbf{k}_c)\cdot\mathbf{E}, \qquad \text{(S30)}
$$

where Eq. (S27) is used. The total energy of the wave packet in the presence of the electric field then becomes

$$
\left\langle\tilde{W}_n(\mathbf{r})\middle|\hat{\mathcal{H}}\middle|\tilde{W}_n(\mathbf{r})\right\rangle = \left\langle\tilde{W}_n(\mathbf{r})\middle|\left[\hat{\mathcal{H}}_c+\mathbf{E}\cdot(\hat{\mathbf{r}}-\mathbf{r}_c)\right]\middle|\tilde{W}_n(\mathbf{r})\right\rangle = \varepsilon_{n\mathbf{k}_c} + e\mathbf{E}\cdot\mathbf{r}_c + \frac{e}{2}\mathbf{E}\cdot\mathbf{G}_n(\mathbf{k}_c)\cdot\mathbf{E}, \qquad \text{(S31)}
$$

where the second term is not observable because $\mathbf{r}_c = -\nabla_{\mathbf{k}}\arg w_n(\mathbf{k})|_{\mathbf{k}=\mathbf{k}_c} + \boldsymbol{\mathcal{A}}_n(\mathbf{k}_c)$ depends on gauge (see Sec. SII B). In fact, this term can be regarded as the electric dipole correction to the wave packet [4]. The wave packet energy should be written as

$$
\tilde{\varepsilon}_{n\mathbf{k}_c} = \varepsilon_{n\mathbf{k}_c} + \frac{e}{2}\mathbf{E}\cdot\mathbf{G}_n(\mathbf{k}_c)\cdot\mathbf{E}, \qquad \text{(S32)}
$$

which is consistent with the electric-field-modified band energy derived from the standard perturbation theory [Eq. (S11)] except that the argument here is $\mathbf{k}_c$ rather than $\mathbf{k}$.

SIII. LUTTINGER-KOHN APPROACH

To extract the electric-field-modified Berry connection and band energy, we now consider a different formalism known as the Luttinger-Kohn approach [5]. To implement the approach, we first rewrite the ansatz [Eq. (S8)] as

$$
\mathcal{H}(\mathbf{k}) = \mathcal{H}_0(\mathbf{k}) + \lambda e\mathbf{E}\cdot\mathrm{i}\nabla_{\mathbf{k}}, \qquad \text{(S33)}
$$

where the parameter λ is introduced to track the order of perturbation and can be conveniently set to unity as needed. We then perform a unitary Schrieffer-Wolff transformation $|u_{n\mathbf{k}}(\mathbf{r})\rangle \to \mathrm{e}^{\lambda\mathcal{S}(\mathbf{k})}|u_{n\mathbf{k}}(\mathbf{r})\rangle$, where the generator $\mathcal{S}(\mathbf{k})$ is anti-Hermitian to guarantee the unitarity of the transformation [6], such that $\mathrm{e}^{\lambda\mathcal{S}(\mathbf{k})}|u_{n\mathbf{k}}(\mathbf{r})\rangle$ diagonalizes $\mathcal{H}(\mathbf{k})$ to the desired order of λ (or, equivalently, $\mathbf{E}$) with an appropriate choice of the generator $\mathcal{S}(\mathbf{k})$. The unitarily transformed Hamiltonian reads

$$
\begin{aligned}
\mathcal{H}_{\text{eff}}(\mathbf{k}) &= \mathrm{e}^{-\lambda\mathcal{S}(\mathbf{k})}\mathcal{H}(\mathbf{k})\mathrm{e}^{\lambda\mathcal{S}(\mathbf{k})} = \mathcal{H}(\mathbf{k}) + \lambda[\mathcal{H}(\mathbf{k}),\mathcal{S}(\mathbf{k})] + \frac{\lambda^2}{2}[[\mathcal{H}(\mathbf{k}),\mathcal{S}(\mathbf{k})],\mathcal{S}(\mathbf{k})] + \cdots \\
&= \mathcal{H}_0(\mathbf{k}) + \lambda e\mathbf{E}\cdot\mathrm{i}\nabla_{\mathbf{k}} + \lambda[\mathcal{H}_0(\mathbf{k}) + \lambda e\mathbf{E}\cdot\mathrm{i}\nabla_{\mathbf{k}},\mathcal{S}(\mathbf{k})] + \frac{\lambda^2}{2}[[\mathcal{H}_0(\mathbf{k}) + \lambda e\mathbf{E}\cdot\mathrm{i}\nabla_{\mathbf{k}},\mathcal{S}(\mathbf{k})],\mathcal{S}(\mathbf{k})] + \cdots \qquad \text{(S34)} \\
&= \mathcal{H}_0(\mathbf{k}) + \lambda\big(e\mathbf{E}\cdot\mathrm{i}\nabla_{\mathbf{k}} + [\mathcal{H}_0(\mathbf{k}),\mathcal{S}(\mathbf{k})]\big) + \lambda^2\left([e\mathbf{E}\cdot\mathrm{i}\nabla_{\mathbf{k}},\mathcal{S}(\mathbf{k})] + \frac{1}{2}[[\mathcal{H}_0(\mathbf{k}),\mathcal{S}(\mathbf{k})],\mathcal{S}(\mathbf{k})]\right) + \mathcal{O}(\lambda^3),
\end{aligned}
$$

where the Baker-Campbell-Hausdorff formula is used. Here, we would expect $\mathrm{e}^{\lambda\mathcal{S}(\mathbf{k})}|u_{n\mathbf{k}}(\mathbf{r})\rangle$ to diagonalize $\mathcal{H}(\mathbf{k})$ to the first order of λ. This is equivalent to that $|u_{n\mathbf{k}}(\mathbf{r})\rangle$ diagonalizes $\mathcal{H}_{\text{eff}}(\mathbf{k})$ to the first order of λ such that the energy correction arising from the third term in Eq. (S34) (scaled as λ^2) coincides with its expectation value in $|u_{n\mathbf{k}}(\mathbf{r})\rangle$. This requires that the second term in Eq. (S34) becomes diagonal in the Hilbert space spanned by $|u_{n\mathbf{k}}(\mathbf{r})\rangle$'s. Consequently, we can write

$$
\langle u_{m\mathbf{k}}(\mathbf{r})|\left\{e\mathbf{E}\cdot\mathrm{i}\nabla_{\mathbf{k}} + [\mathcal{H}_0(\mathbf{k}),\mathcal{S}(\mathbf{k})]\right\}|u_{n\mathbf{k}}(\mathbf{r})\rangle = e\mathbf{E}\cdot\langle u_{m\mathbf{k}}(\mathbf{r})|\mathrm{i}\nabla_{\mathbf{k}}|u_{n\mathbf{k}}(\mathbf{r})\rangle + (\varepsilon_{m\mathbf{k}}-\varepsilon_{n\mathbf{k}})\langle u_{m\mathbf{k}}(\mathbf{r})|\mathcal{S}(\mathbf{k})|u_{n\mathbf{k}}(\mathbf{r})\rangle = 0, \qquad \text{(S35)}
$$

where $m\neq n$. Equation (S35) is referred to as the Luttinger-Kohn condition [5, 6], with which the generator of the Schrieffer-Wolff transformation can be solved as

$$
\mathcal{S}_{mn}(\mathbf{k}) = \frac{e\mathbf{E}\cdot\boldsymbol{\mathcal{A}}_{mn}(\mathbf{k})}{\varepsilon_{n\mathbf{k}}-\varepsilon_{m\mathbf{k}}}. \qquad \text{(S36)}
$$

The anti-Hermicity of $\mathcal{S}(\mathbf{k})$ can be checked as $\mathcal{S}^*_{nm}(\mathbf{k}) = \frac{e\mathbf{E}\cdot\boldsymbol{\mathcal{A}}^*_{nm}(\mathbf{k})}{\varepsilon_{m\mathbf{k}}-\varepsilon_{n\mathbf{k}}} = \frac{e\mathbf{E}\cdot\boldsymbol{\mathcal{A}}_{mn}(\mathbf{k})}{\varepsilon_{m\mathbf{k}}-\varepsilon_{n\mathbf{k}}} = -\mathcal{S}_{mn}(\mathbf{k})$, where $\boldsymbol{\mathcal{A}}^*_{nm}(\mathbf{k}) = \boldsymbol{\mathcal{A}}_{nm}(\mathbf{k})$ is used. We mention that the diagonal entries of $\mathcal{S}(\mathbf{k})$ cannot be uniquely determined, as the anti-Hermicity only requires the real parts of them to vanish. Such entries are set to zero in Ref. [7], but their specific values would not affect the calculation of the electric-field-modified Berry connection and band energy. To see this, we first write down the eigenenergy of $\mathcal{H}(\mathbf{k})$, which coincides with the expectation value of $\mathcal{H}_{\text{eff}}(\mathbf{k})$, as

$$\begin{aligned}
\langle u_{n\mathbf{k}}(\mathbf{r})|\mathcal{H}_{\text{eff}}(\mathbf{k})\,|u_{n\mathbf{k}}(\mathbf{r})\rangle &= \varepsilon_{n\mathbf{k}} + \lambda e\mathbf{E}\cdot\boldsymbol{\mathcal{A}}_n(\mathbf{k}) + \lambda^2\left\langle u_{n\mathbf{k}}(\mathbf{r})\middle|\left([e\mathbf{E}\cdot\mathrm{i}\nabla_{\mathbf{k}},\mathcal{S}(\mathbf{k})] + \frac{1}{2}[[\mathcal{H}_0(\mathbf{k}),\mathcal{S}(\mathbf{k})],\mathcal{S}(\mathbf{k})]\right)\middle|u_{n\mathbf{k}}(\mathbf{r})\right\rangle \\
&= \varepsilon_{n\mathbf{k}} + \lambda e\mathbf{E}\cdot\boldsymbol{\mathcal{A}}_n(\mathbf{k}) + \lambda^2\langle u_{n\mathbf{k}}(\mathbf{r})|\,[e\mathbf{E}\cdot\mathrm{i}\nabla_{\mathbf{k}},\mathcal{S}(\mathbf{k})]\,|u_{n\mathbf{k}}(\mathbf{r})\rangle + \lambda^2\langle u_{n\mathbf{k}}(\mathbf{r})|\,[\varepsilon_{n\mathbf{k}}\mathcal{S}^2(\mathbf{k}) - \mathcal{S}(\mathbf{k})\mathcal{H}_0(\mathbf{k})\mathcal{S}(\mathbf{k})]\,|u_{n\mathbf{k}}(\mathbf{r})\rangle \\
&= \varepsilon_{n\mathbf{k}} + \lambda e\mathbf{E}\cdot\boldsymbol{\mathcal{A}}_n(\mathbf{k}) + \lambda^2\langle u_{n\mathbf{k}}(\mathbf{r})|\,[e\mathbf{E}\cdot\mathrm{i}\nabla_{\mathbf{k}},\mathcal{S}(\mathbf{k})]\,|u_{n\mathbf{k}}(\mathbf{r})\rangle + \lambda^2\sum_{m\neq n}(\varepsilon_{n\mathbf{k}}-\varepsilon_{m\mathbf{k}})S_{nm}(\mathbf{k})S_{mn}(\mathbf{k}) \\
&= \varepsilon_{n\mathbf{k}} + \lambda e\mathbf{E}\cdot\boldsymbol{\mathcal{A}}_n(\mathbf{k}) + \lambda^2\langle u_{n\mathbf{k}}(\mathbf{r})|\,[e\mathbf{E}\cdot\mathrm{i}\nabla_{\mathbf{k}},\mathcal{S}(\mathbf{k})]\,|u_{n\mathbf{k}}(\mathbf{r})\rangle - \lambda^2\sum_{m\neq n}e\mathbf{E}\cdot\boldsymbol{\mathcal{A}}_{nm}(\mathbf{k})S_{mn}(\mathbf{k}) \\
&= \varepsilon_{n\mathbf{k}} + \lambda e\mathbf{E}\cdot\boldsymbol{\mathcal{A}}_n(\mathbf{k}) + \lambda^2\sum_{m\neq n}[e\mathbf{E}\cdot\boldsymbol{\mathcal{A}}_{nm}(\mathbf{k})S_{mn}(\mathbf{k}) - S_{nm}(\mathbf{k})\boldsymbol{\mathcal{A}}_{mn}(\mathbf{k})\cdot e\mathbf{E}] - \lambda^2\sum_{m\neq n}e\mathbf{E}\cdot\boldsymbol{\mathcal{A}}_{nm}(\mathbf{k})S_{mn}(\mathbf{k}) \\
&= \varepsilon_{n\mathbf{k}} + \lambda e\mathbf{E}\cdot\boldsymbol{\mathcal{A}}_n(\mathbf{k}) + \lambda^2 e^2\sum_{m\neq n}\frac{\mathbf{E}\cdot\boldsymbol{\mathcal{A}}_{nm}(\mathbf{k})}{\varepsilon_{n\mathbf{k}}-\varepsilon_{m\mathbf{k}}}\boldsymbol{\mathcal{A}}_{mn}(\mathbf{k})\cdot\mathbf{E}.
\end{aligned} \tag{S37}$$

The electric-field-modified band energy is extracted as the gauge invariant part of $\langle u_{n\mathbf{k}}(\mathbf{r})|\mathcal{H}_{\text{eff}}(\mathbf{k})\,|u_{n\mathbf{k}}(\mathbf{r})\rangle$ when setting $\lambda = 1$. Explicitly, it reads

$$\begin{aligned}
\tilde{\varepsilon}_{n\mathbf{k}} &= \varepsilon_{n\mathbf{k}} + e^2\sum_{m\neq n}\frac{\mathbf{E}\cdot\boldsymbol{\mathcal{A}}_{nm}(\mathbf{k})}{\varepsilon_{n\mathbf{k}}-\varepsilon_{m\mathbf{k}}}\boldsymbol{\mathcal{A}}_{mn}(\mathbf{k})\cdot\mathbf{E} = \varepsilon_{n\mathbf{k}} + e^2\mathbf{E}\cdot\mathrm{Re}\sum_{m\neq n}\frac{\boldsymbol{\mathcal{A}}_{nm}(\mathbf{k})\boldsymbol{\mathcal{A}}_{mn}(\mathbf{k})}{\varepsilon_{n\mathbf{k}}-\varepsilon_{m\mathbf{k}}}\cdot\mathbf{E} \\
&= \varepsilon_{n\mathbf{k}} + \frac{e}{2}\mathbf{E}\cdot\mathbf{G}_n(\mathbf{k})\cdot\mathbf{E}.
\end{aligned} \tag{S38}$$

Equation (S38) is identical to the electric-field-modified band energy derived by the standard perturbation theory [Eq. (S11)].

We now turn to evaluate the electric-field-modified Berry connection. With the Schrieffer-Wolff transformed unit-cell-periodic state $\mathrm{e}^{\mathcal{S}(\mathbf{k})}|u_{n\mathbf{k}}(\mathbf{r})\rangle$, where we set $\lambda = 1$ again, the electric-field-modified Berry connection reads

$$\begin{aligned}
\tilde{\boldsymbol{\mathcal{A}}}_n(\mathbf{k}) &= \langle u_{n\mathbf{k}}(\mathbf{r})|\mathrm{e}^{-\mathcal{S}(\mathbf{k})}\mathrm{i}\nabla_{\mathbf{k}}\mathrm{e}^{\mathcal{S}(\mathbf{k})}|u_{n\mathbf{k}}(\mathbf{r})\rangle \simeq \langle u_{n\mathbf{k}}(\mathbf{r})|\{\mathrm{i}\nabla_{\mathbf{k}} + [\mathrm{i}\nabla_{\mathbf{k}},\mathcal{S}(\mathbf{k})]\}|u_{n\mathbf{k}}(\mathbf{r})\rangle \\
&= \boldsymbol{\mathcal{A}}_n(\mathbf{k}) + \sum_{m\neq n}[\boldsymbol{\mathcal{A}}_{nm}(\mathbf{k})\mathcal{S}_{mn}(\mathbf{k}) - \mathcal{S}_{nm}(\mathbf{k})\boldsymbol{\mathcal{A}}_{mn}(\mathbf{k})] = \boldsymbol{\mathcal{A}}_n(\mathbf{k}) + 2\,\mathrm{Re}\sum_{m\neq n}\boldsymbol{\mathcal{A}}_{nm}(\mathbf{k})\mathcal{S}_{mn}(\mathbf{k}) \\
&= \boldsymbol{\mathcal{A}}_n(\mathbf{k}) + 2e\mathrm{Re}\sum_{m\neq n}\frac{\boldsymbol{\mathcal{A}}_{nm}(\mathbf{k})\boldsymbol{\mathcal{A}}_{mn}(\mathbf{k})}{\varepsilon_{n\mathbf{k}}-\varepsilon_{m\mathbf{k}}}\cdot\mathbf{E} = \boldsymbol{\mathcal{A}}_n(\mathbf{k}) + \mathbf{G}_n(\mathbf{k})\cdot\mathbf{E}.
\end{aligned} \tag{S39}$$

Equation (S39) is identical to the electric-field-modified Berry connection derived by the standard perturbation theory [Eq. (S6)].

SIV. NONLINEAR TRANSPORT

We now evaluate the second-order nonlinear transport. As discussed in the main text, this requires expanding the velocity and non-equilibrium distribution function to the second order of $\mathbf{E}$. In this section, we will first acquire the expansions and then derive the resulting nonlinear conductivity.

A. Velocity

The velocity comprises a drift term associated with the electric-field-modified band energy $\tilde{\varepsilon}_{n\mathbf{k}}$ and an anomalous term associated with the electric-field-modified Berry curvature $\tilde{\boldsymbol{\Omega}}_n(\mathbf{k}) = \nabla_{\mathbf{k}}\times\tilde{\boldsymbol{\mathcal{A}}}_n(\mathbf{k})$ [8]. Explicitly, the velocity

associated with the nth energy band reads

$$\begin{aligned}\tilde{\mathbf{v}}_n(\mathbf{k}) &= \frac{1}{\hbar}\nabla_{\mathbf{k}}\tilde{\varepsilon}_{n\mathbf{k}} - \dot{\mathbf{k}}\times\tilde{\mathbf{\Omega}}_n(\mathbf{k}) = \frac{1}{\hbar}\nabla_{\mathbf{k}}\left[\varepsilon_{n\mathbf{k}} + \frac{e}{2}\mathbf{E}\cdot\mathbf{G}_n(\mathbf{k})\cdot\mathbf{E}\right] + \frac{e}{\hbar}\mathbf{E}\times\{\nabla_{\mathbf{k}}\times[\boldsymbol{\mathcal{A}}_n(\mathbf{k}) + \mathbf{G}_n(\mathbf{k})\cdot\mathbf{E}]\} \\ &= \frac{1}{\hbar}\nabla_{\mathbf{k}}\varepsilon_{n\mathbf{k}} + \frac{e}{\hbar}\mathbf{E}\times\mathbf{\Omega}_n(\mathbf{k}) + \frac{e}{2\hbar}\nabla_{\mathbf{k}}[\mathbf{E}\cdot\mathbf{G}_n(\mathbf{k})\cdot\mathbf{E}] + \frac{e}{\hbar}\mathbf{E}\times\{\nabla_{\mathbf{k}}\times[\mathbf{G}_n(\mathbf{k})\cdot\mathbf{E}]\},\end{aligned} \tag{S40}$$

where we have used the equation of motion $\hbar\dot{\mathbf{k}} = -e\mathbf{E}$. For transparency, it is more convenient to express $\tilde{\mathbf{v}}_n(\mathbf{k})$ in the component form as

$$\begin{aligned}\tilde{v}_n^i &= \frac{1}{\hbar}\partial_i\varepsilon_n + \frac{e}{\hbar}\epsilon_{ijk}E_j\Omega_n^k + \frac{e}{2\hbar}\partial_i G_n^{jk}E_jE_k + \frac{e}{\hbar}\epsilon_{ijk}E_j\epsilon_{klr}\partial_l G_n^{rs}E_s \\ &= \frac{1}{\hbar}\partial_i\varepsilon_n + \frac{e}{\hbar}\epsilon_{ijk}\Omega_n^k E_j + \frac{e}{2\hbar}\partial_i G_n^{jk}E_jE_k + \frac{e}{\hbar}(\delta_{il}\delta_{jr} - \delta_{ir}\delta_{jl})\partial_l G_n^{rs}E_jE_s \\ &= \frac{1}{\hbar}\partial_i\varepsilon_n + \frac{e}{\hbar}\epsilon_{ijk}\Omega_n^k E_j + \frac{e}{2\hbar}\partial_i G_n^{jk}E_jE_k + \frac{e}{\hbar}\left(\partial_i G_n^{js} - \partial_j G_n^{is}\right)E_jE_s \\ &= \frac{1}{\hbar}\partial_i\varepsilon_n + \frac{e}{\hbar}\epsilon_{ijk}\Omega_n^k E_j + \frac{e}{2\hbar}\left[3\partial_i G_n^{jk} - \left(\partial_j G_n^{ik} + \partial_k G_n^{ij}\right)\right]E_jE_k \\ &= \frac{1}{\hbar}\partial_i\varepsilon_n + \frac{e}{\hbar}\Omega_n^{ij}E_j + \frac{e}{2\hbar}\left[3\partial_i G_n^{jk} - \left(\partial_j G_n^{ik} + \partial_k G_n^{ij}\right)\right]E_jE_k \\ &= \frac{1}{\hbar}\partial_i\varepsilon_n + \frac{e}{2\hbar}(\Omega_n^{ij}E_j + \Omega_n^{ik}E_k) + \frac{e}{2\hbar}\left[3\partial_i G_n^{jk} - \left(\partial_j G_n^{ik} + \partial_k G_n^{ij}\right)\right]E_jE_k,\end{aligned} \tag{S41}$$

where we have omitted the argument $\mathbf{k}$ in variables to circumvent misguidance in subscripts/superscripts (e.g., $\varepsilon_{n\mathbf{k}} \to \varepsilon_n$ and $\partial_{k_i} \to \partial_i$) and we have also symmetrized indices $\{j,k\}$ (e.g., by defining the Berry curvature tensor $\Omega_n^{ij} = \epsilon_{ijk}\Omega_n^k = \partial_i\mathcal{A}_n^j - \partial_j\mathcal{A}_n^i$).

B. Boltzmann Formalism

The non-equilibrium distribution function $f(\mathbf{r},\mathbf{k},t)$ can be found using the Boltzmann formalism. In the phase space, the non-equilibrium distribution function is governed by [9]

$$\frac{\partial f}{\partial t} + \dot{\mathbf{r}}\cdot\nabla_{\mathbf{r}}f + \dot{\mathbf{k}}\cdot\nabla_{\mathbf{k}}f = \mathcal{I}\{f\}, \tag{S42}$$

where $\mathcal{I}\{f\}$ is the collision integral. For simplicity, we focus on the stationary case such that f does not vary with time, i.e., $f = f(\mathbf{r},\mathbf{k})$. Moreover, for a weak applied electric field, it is legitimate to assume that f is approximately uniform, resulting in a further simplification $f = f(\mathbf{k})$. In the relaxation time approximation, the collision integral can be written as $-[f(\mathbf{k}) - f_0(\tilde{\varepsilon}_{n\mathbf{k}})]/\tau$, where we assume a constant relaxation time τ for simplicity and f_0 is the Fermi-Dirac distribution function. Consequently, Eq. (S42) is reduced to

$$\dot{\mathbf{k}}\cdot\nabla_{\mathbf{k}}f(\mathbf{k}) = -\frac{f(\mathbf{k}) - f_0(\tilde{\varepsilon}_{n\mathbf{k}})}{\tau}. \tag{S43}$$

Making use of the equation of motion $\hbar\dot{\mathbf{k}} = -e\mathbf{E}$, the non-equilibrium distribution function admits a power series solution

$$f(\mathbf{k}) = \left(1 - \frac{e\tau}{\hbar}\mathbf{E}\cdot\nabla_{\mathbf{k}}\right)^{-1} f_0(\tilde{\varepsilon}_{n\mathbf{k}}) = \sum_{\nu=0}^{\infty}\left(\frac{e\tau}{\hbar}\mathbf{E}\cdot\nabla_{\mathbf{k}}\right)^{\nu} f_0(\tilde{\varepsilon}_{n\mathbf{k}}). \tag{S44}$$

Plugging Eq. (S11) into Eq. (S44), the non-equilibrium distribution function, to the second order of $\mathbf{E}$, reads

$$f(\mathbf{k}) = f_0(\varepsilon_{n\mathbf{k}}) + \frac{e}{2}\mathbf{E}\cdot\mathbf{G}_n(\mathbf{k})\cdot\mathbf{E}\left.\frac{\partial f_0(\varepsilon)}{\partial\varepsilon}\right|_{\varepsilon=\varepsilon_{n\mathbf{k}}} + \frac{e\tau}{\hbar}\mathbf{E}\cdot\nabla_{\mathbf{k}}f_0(\varepsilon_{n\mathbf{k}}) + \left(\frac{e\tau}{\hbar}\mathbf{E}\cdot\nabla_{\mathbf{k}}\right)^2 f_0(\varepsilon_{n\mathbf{k}}). \tag{S45}$$

In a more compact form, we rewrite the non-equilibrium distribution function as

$$f = f_0 + \frac{e}{2}G_n^{ij}E_iE_jf_0' + \frac{e\tau}{\hbar}E_i\partial_i f_0 + \frac{e^2\tau^2}{\hbar^2}E_iE_j\partial_i\partial_j f_0, \tag{S46}$$

where we use the notations $f = f(\mathbf{k})$, $f_0 = f_0(\varepsilon_{n\mathbf{k}})$, and $f_0' = \frac{\partial f_0(\varepsilon)}{\partial\varepsilon}|_{\varepsilon=\varepsilon_{n\mathbf{k}}}$.

C. Nonlinear Conductivity

With the velocity and the non-equilibrium distribution function in hand, it is straightforward to calculate the nonlinear conductivity. For this purpose, we first evaluate the current density

$$\mathbf{J} = \frac{-e}{\mathcal{V}} \sum_n \sum_{\mathbf{k}} \tilde{\mathbf{v}}_n(\mathbf{k}) f(\mathbf{k}) = -e \sum_n \int [d\mathbf{k}] \tilde{\mathbf{v}}_n(\mathbf{k}) f(\mathbf{k}), \tag{S47}$$

where $\mathcal{V}$ is the volume of the system and $[d\mathbf{k}] \equiv d^d\mathbf{k}/(2\pi)^d$ with dimension d. As we have respectively found $\tilde{\mathbf{v}}_n(\mathbf{k})$ and $f(\mathbf{k})$ to the second order of $\mathbf{E}$ in Secs. SIV A and SIV B, Eq. (S47) can also be evaluated to the second order of $\mathbf{E}$. In the component form, the current density can thus be written as

$$J_i \simeq \sigma_{ij} E_j + \sigma_{ijk} E_j E_k, \tag{S48}$$

where σ_{ij} is the linear conductivity and σ_{ijk} is the second-order nonlinear conductivity. In the rest of this section, we will focus on the latter. It is worth noting that the nonlinear transport can be divided into three categories according to the dependence on the relaxation time τ. First, the τ^2-dependent nonlinear current reads

$$J_i^{\mathrm{d}} = -e \sum_n \int [d\mathbf{k}] v_n^i \frac{e^2\tau^2}{\hbar^2} E_j E_k \partial_j \partial_k f_0 = -\frac{\tau^2 e^3}{\hbar^3} \sum_n \int [d\mathbf{k}] (\partial_i \varepsilon_n) E_j E_k \partial_j \partial_k f_0 = -\frac{e^3\tau^2}{\hbar^3} \sum_n \int [d\mathbf{k}] \big(\partial_i \partial_j \partial_k \varepsilon_n\big) f_0 E_j E_k, \tag{S49}$$

which is associated with the Drude mechanism. Second, the τ-dependent nonlinear current reads

$$\begin{aligned} J_i^{\mathrm{bc}} &= -e \sum_n \int [d\mathbf{k}] \frac{e}{2\hbar} (\Omega_n^{ij} E_j + \Omega_n^{ik} E_k) \frac{e\tau}{\hbar} E_l \partial_l f_0 = -\frac{e^3\tau}{2\hbar^2} \sum_n \int [d\mathbf{k}] (\Omega_n^{ij} E_j E_k \partial_k f_0 + \Omega_n^{ik} E_k E_j \partial_j f_0) \\ &= \frac{e^3\tau}{2\hbar^2} \sum_n \int [d\mathbf{k}] (\partial_k \Omega_n^{ij} + \partial_j \Omega_n^{ik}) f_0 E_j E_k, \end{aligned} \tag{S50}$$

which is referred to as the Berry curvature dipole contribution. Lastly, the τ-free (i.e., intrinsic) nonlinear current reads

$$\begin{aligned} J_i^{\mathrm{qm}} &= -e \sum_n \int [d\mathbf{k}] v_n^i \frac{e}{2} G_n^{jk} E_j E_k f_0' - e \sum_n \int [d\mathbf{k}] \frac{e}{2\hbar} \left[3\partial_i G_n^{jk} - \big(\partial_j G_n^{ik} + \partial_k G_n^{ij}\big)\right] E_j E_k f_0 \\ &= -\frac{e^2}{2\hbar} \sum_n \int [d\mathbf{k}] (\partial_i \varepsilon_n) G_n^{jk} E_j E_k f_0' - \frac{e^2}{2\hbar} \sum_n \int [d\mathbf{k}] \left[3\partial_i G_n^{jk} - \big(\partial_j G_n^{ik} + \partial_k G_n^{ij}\big)\right] E_j E_k f_0 \\ &= \frac{e^2}{2\hbar} \sum_n \int [d\mathbf{k}] (\partial_i G_n^{jk}) E_j E_k f_0 - \frac{e^2}{2\hbar} \sum_n \int [d\mathbf{k}] \left[3\partial_i G_n^{jk} - \big(\partial_j G_n^{ik} + \partial_k G_n^{ij}\big)\right] E_j E_k f_0 \\ &= -\frac{e^2}{\hbar} \sum_n \int [d\mathbf{k}] \left[\partial_i G_n^{jk} - \frac{1}{2} \big(\partial_j G_n^{ik} + \partial_k G_n^{ij}\big)\right] f_0 E_j E_k. \end{aligned} \tag{S51}$$

which is known as the quantum metric dipole contribution and will be further demonstrated in the following [see Eq. (S60)].

The nonlinear conductivities associated with the Drude, Berry curvature dipole, and quantum metric dipole mechanisms can be respectively read off from Eqs. (S49), (S50), and (S51) as

$$\sigma_{ijk}^{\mathrm{d}} = -\frac{e^3\tau^2}{\hbar^3} \sum_n \int [d\mathbf{k}] \big(\partial_i \partial_j \partial_k \varepsilon_n\big) f_0, \tag{S52}$$

$$\sigma_{ijk}^{\mathrm{bc}} = \frac{e^3\tau}{2\hbar^2} \sum_n \int [d\mathbf{k}] \big(\partial_k \Omega_n^{ij} + \partial_j \Omega_n^{ik}\big) f_0, \tag{S53}$$

$$\sigma_{ijk}^{\mathrm{qm}} = -\frac{e^2}{\hbar} \sum_n \int [d\mathbf{k}] \left[\partial_i G_n^{jk} - \frac{1}{2} \big(\partial_k G_n^{ij} + \partial_j G_n^{ik}\big)\right] f_0, \tag{S54}$$

which correspond to Eqs. (11)-(13) in the main text. Alternatively, through integrating by parts, these expressions can be rewritten as

$$\sigma_{ijk}^{\mathrm{d}} = \frac{e^3\tau^2}{\hbar^2} \sum_n \int [d\mathbf{k}] v_n^i \big(\partial_j \partial_k \varepsilon_n\big) f_0', \tag{S55}$$

$$\sigma^{\rm bc}_{ijk} = -\frac{e^3\tau}{2\hbar}\sum_n \int [d\mathbf{k}] \left(v^k_n\Omega^{ij}_n + v^j_n\Omega^{ik}_n\right) f_0', \tag{S56}$$

$$\sigma^{\rm qm}_{ijk} = e^2 \sum_n \int [d\mathbf{k}] \left[v^i_n G^{jk}_n - \frac{1}{2}\left(v^k_n G^{ij}_n + v^j_n G^{ik}_n\right)\right] f_0'. \tag{S57}$$

The derived quantum-metric-induced nonlinear conductivity [Eqs. (S54) and (S57)] is consistent with those listed in Refs. [10–12], which are not symmetrized for indices $\{j,k\}$. To better illustrate the quantum metric origin of $\sigma^{\rm qm}_{ijk}$, we rewrite Eqs. (S54) and (S57) as

$$\sigma^{\rm qm}_{ijk} = -\frac{e^3}{\hbar}\sum_n \int [d\mathbf{k}] \left[\partial_i \mathcal{G}^{jk}_n - \frac{1}{2}\left(\partial_k \mathcal{G}^{ij}_n + \partial_j \mathcal{G}^{ik}_n\right)\right] f_0, \tag{S58}$$

$$\sigma^{\rm qm}_{ijk} = e^3 \sum_n \int [d\mathbf{k}] \left[v^i_n \mathcal{G}^{jk}_n - \frac{1}{2}\left(v^k_n \mathcal{G}^{ij}_n + v^j_n \mathcal{G}^{ik}_n\right)\right] f_0', \tag{S59}$$

where $\boldsymbol{\mathcal{G}}_n(\mathbf{k}) = 2\,\mathrm{Re}\sum_{m\neq n} \boldsymbol{\mathcal{A}}_{mn}(\mathbf{k})\boldsymbol{\mathcal{A}}_{mn}(\mathbf{k})/(\varepsilon_{n\mathbf{k}} - \varepsilon_{m\mathbf{k}})$ is the band-normalized quantum metric, differing from the Berry connection polarizability $\mathbf{G}_n(\mathbf{k})$ by a factor of e. The kernel of Eq. (S59) reads

$$\Lambda^{ijk}_n(\mathbf{k}) = v^i_n \mathcal{G}^{jk}_n - \frac{1}{2}\left(v^k_n \mathcal{G}^{ij}_n + v^j_n \mathcal{G}^{ik}_n\right), \tag{S60}$$

which is referred to as the "quantum metric dipole" as the band-normalized quantum metric $\boldsymbol{\mathcal{G}}_n(\mathbf{k})$ is related to the quantum metric $\mathbf{g}_n(\mathbf{k}) = \mathrm{Re}\sum_{m\neq n} \boldsymbol{\mathcal{A}}_{nm}(\mathbf{k})\boldsymbol{\mathcal{A}}_{mn}(\mathbf{k})$ through $\boldsymbol{\mathcal{G}}_n(\mathbf{k}) = -\partial \mathbf{g}_n(\mathbf{k})/\partial\varepsilon_{n\mathbf{k}}$ [13].

---

[1] Consider a gauge transformation $|u_{n\mathbf{k}}(\mathbf{r})\rangle \to e^{\mathrm{i}\phi_{n\mathbf{k}}}|u_{n\mathbf{k}}(\mathbf{r})\rangle$. The resulting transformed interband Berry connection becomes $\boldsymbol{\mathcal{A}}_{mn}(\mathbf{k}) \to \langle u_{m\mathbf{k}}(\mathbf{r})|e^{-\mathrm{i}\phi_{m\mathbf{k}}}(\mathrm{i}\nabla_{\mathbf{k}}e^{\mathrm{i}\phi_{n\mathbf{k}}})|u_{n\mathbf{k}}(\mathbf{r})\rangle + \langle u_{m\mathbf{k}}(\mathbf{r})|e^{-\mathrm{i}\phi_{m\mathbf{k}}}e^{\mathrm{i}\phi_{n\mathbf{k}}}\mathrm{i}\nabla_{\mathbf{k}}|u_{n\mathbf{k}}(\mathbf{r})\rangle = e^{\mathrm{i}(\phi_{n\mathbf{k}}-\phi_{m\mathbf{k}})}\boldsymbol{\mathcal{A}}_{mn}(\mathbf{k})$, where we have used $\langle u_{m\mathbf{k}}(\mathbf{r})|u_{n\mathbf{k}}(\mathbf{r})\rangle = 0$ for $m \neq n$. Consequently, $\boldsymbol{\mathcal{A}}_{nm}(\mathbf{k})\boldsymbol{\mathcal{A}}_{mn}(\mathbf{k})$ possesses gauge invariance, which is inherited by the Berry connection polarizability.

[2] R. Resta, Quantum-Mechanical Position Operator in Extended Systems, Phys. Rev. Lett. **80**, 1800 (1998).

[3] R. W. Nunes and X. Gonze, Berry-phase treatment of the homogeneous electric field perturbation in insulators, Phys. Rev. B **63**, 155107 (2001).

[4] Y. Gao, Semiclassical dynamics and nonlinear charge current, Front. Phys. **14**, 33404 (2019).

[5] J. M. Luttinger and W. Kohn, Motion of Electrons and Holes in Perturbed Periodic Fields, Phys. Rev. **97**, 869 (1955).

[6] J. R. Schrieffer and P. A. Wolff, Relation between the Anderson and Kondo Hamiltonians, Phys. Rev. **149**, 491 (1966).

[7] D. Kaplan, T. Holder, and B. Yan, Unification of Nonlinear Anomalous Hall Effect and Nonreciprocal Magnetoresistance in Metals by the Quantum Geometry, Phys. Rev. Lett. **132**, 026301 (2024).

[8] D. Xiao, M.-C. Chang, and Q. Niu, Berry phase effects on electronic properties, Rev. Mod. Phys. **82**, 1959 (2010).

[9] N. W. Ashcroft and N. D. Mermin, *Solid State Physics* (Saunders College, Philadelphia, 1976).

[10] Y. Gao, S. A. Yang, and Q. Niu, Field Induced Positional Shift of Bloch Electrons and Its Dynamical Implications, Phys. Rev. Lett. **112**, 166601 (2014).

[11] C. Wang, Y. Gao, and D. Xiao, Intrinsic Nonlinear Hall Effect in Antiferromagnetic Tetragonal CuMnAs, Phys. Rev. Lett. **127**, 277201 (2021).

[12] H. Liu, J. Zhao, Y.-X. Huang, W. Wu, X.-L. Sheng, C. Xiao, and S. A. Yang, Intrinsic Second-Order Anomalous Hall Effect and Its Application in Compensated Antiferromagnets, Phys. Rev. Lett. **127**, 277202 (2021).

[13] To see how the band-normalized quantum metric $\boldsymbol{\mathcal{G}}_n(\mathbf{k})$ is related to the quantum metric $\mathbf{g}_n(\mathbf{k})$, it would be helpful to first rewrite $\boldsymbol{\mathcal{G}}_n(\mathbf{k})$ and $\mathbf{g}_n(\mathbf{k})$. We note that $\langle u_{m\mathbf{k}}(\mathbf{r})|\mathcal{H}_0(\mathbf{k})|u_{n\mathbf{k}}(\mathbf{r})\rangle = 0$ for $m \neq n$. This implies $\langle \nabla_{\mathbf{k}} u_{m\mathbf{k}}(\mathbf{r})|\mathcal{H}_0(\mathbf{k})|u_{n\mathbf{k}}(\mathbf{r})\rangle + \langle u_{m\mathbf{k}}(\mathbf{r})|\mathcal{H}_0(\mathbf{k})|\nabla_{\mathbf{k}} u_{n\mathbf{k}}(\mathbf{r})\rangle + \langle u_{m\mathbf{k}}(\mathbf{r})|\nabla_{\mathbf{k}}\mathcal{H}_0(\mathbf{k})|u_{n\mathbf{k}}(\mathbf{r})\rangle = (\varepsilon_{m\mathbf{k}} - \varepsilon_{n\mathbf{k}})\langle u_{m\mathbf{k}}(\mathbf{r})|\nabla_{\mathbf{k}}|u_{n\mathbf{k}}(\mathbf{r})\rangle + \langle u_{m\mathbf{k}}(\mathbf{r})|\nabla_{\mathbf{k}}\mathcal{H}_0(\mathbf{k})|u_{n\mathbf{k}}(\mathbf{r})\rangle = 0$, from which we obtain $\boldsymbol{\mathcal{A}}_{mn}(\mathbf{k}) = \mathrm{i}\langle u_{m\mathbf{k}}(\mathbf{r})|\nabla_{\mathbf{k}}\mathcal{H}_0(\mathbf{k})|u_{n\mathbf{k}}(\mathbf{r})\rangle/(\varepsilon_{n\mathbf{k}} - \varepsilon_{m\mathbf{k}})$. Consequently, we can rewrite $\mathbf{g}_n(\mathbf{k}) = \mathrm{Re}\sum_{m\neq n}\langle u_{n\mathbf{k}}(\mathbf{r})|\nabla_{\mathbf{k}}\mathcal{H}_0(\mathbf{k})|u_{m\mathbf{k}}(\mathbf{r})\rangle\langle u_{m\mathbf{k}}(\mathbf{r})|\nabla_{\mathbf{k}}\mathcal{H}_0(\mathbf{k})|u_{n\mathbf{k}}(\mathbf{r})\rangle/(\varepsilon_{n\mathbf{k}} - \varepsilon_{m\mathbf{k}})^2$ and $\boldsymbol{\mathcal{G}}_n(\mathbf{k}) = 2\,\mathrm{Re}\sum_{m\neq n}\langle u_{n\mathbf{k}}(\mathbf{r})|\nabla_{\mathbf{k}}\mathcal{H}_0(\mathbf{k})|u_{m\mathbf{k}}(\mathbf{r})\rangle\langle u_{m\mathbf{k}}(\mathbf{r})|\nabla_{\mathbf{k}}\mathcal{H}_0(\mathbf{k})|u_{n\mathbf{k}}(\mathbf{r})\rangle/(\varepsilon_{n\mathbf{k}} - \varepsilon_{m\mathbf{k}})^3$. It is then straightforward to check that $\boldsymbol{\mathcal{G}}_n(\mathbf{k}) = -\partial\mathbf{g}_n(\mathbf{k})/\partial\varepsilon_{n\mathbf{k}}$.
